# Drug Binding Poses Relate Structure with Efficacy in the μ Opioid Receptor

**DOI:** 10.1016/j.jmb.2017.05.009

**Published:** 2017-06-16

**Authors:** Katy J. Sutcliffe, Graeme Henderson, Eamonn Kelly, Richard B. Sessions

**Affiliations:** 1 School of Physiology, Pharmacology and Neuroscience, University of Bristol, Bristol BS8 1TD, UK; 2 School of Biochemistry, University of Bristol, Bristol BS8 1TD, UK

**Keywords:** MOPr, μ-opioid receptor, GPCR, G protein-coupled receptor, TM, transmembrane domain, BRET, bioluminescence resonance energy transfer, MD, molecular dynamics, HEK 293, human embryonic kidney 293, Rluc, renilla luciferase, GFP, green fluorescent protein, YFP, yellow fluorescent protein, cMD, conventional MD, aMD, accelerated MD, β-FNA, β-funaltrexamine, PC, principal component, PCA, principal component analysis, DMEM, Dulbecco's modified Eagle's medium, G protein-coupled receptor, μ-opioid receptor, molecular dynamics, buprenorphine

## Abstract

The μ-opioid receptor (MOPr) is a clinically important G protein-coupled receptor that couples to G_i/o_ proteins and arrestins. At present, the receptor conformational changes that occur following agonist binding and activation are poorly understood. This study has employed molecular dynamics simulations to investigate the binding mode and receptor conformational changes induced by structurally similar opioid ligands of widely differing intrinsic agonist efficacy, norbuprenorphine, buprenorphine, and diprenorphine. Bioluminescence resonance energy transfer assays for G_i_ activation and arrestin-3 recruitment in human embryonic kidney 293 cells confirmed that norbuprenorphine is a high efficacy agonist, buprenorphine a low efficacy agonist, and diprenorphine an antagonist at the MOPr. Molecular dynamics simulations revealed that these ligands adopt distinct binding poses and engage different subsets of residues, despite sharing a common morphinan scaffold. Notably, norbuprenorphine interacted with sodium ion-coordinating residues W293^6.48^ and N150^3.35^, whilst buprenorphine and diprenorphine did not. Principal component analysis of the movements of the receptor transmembrane domains showed that the buprenorphine-bound receptor occupied a distinct set of conformations to the norbuprenorphine-bound receptor. Addition of an allosteric sodium ion caused the receptor and ligand to adopt an inactive conformation. The differences in ligand–residue interactions and receptor conformations observed here may underlie the differing efficacies for cellular signalling outputs for these ligands.

## Highlights

• Efficacy at the MOPr is explored with molecular dynamics simulations.
• Ligands of different efficacy have distinct binding poses and residue interactions.
• High and low efficacy ligands induce different conformations of receptor helices.
• This provides an explanation for the different signalling abilities of these ligands.

## Graphical Abstract

**Figure:**
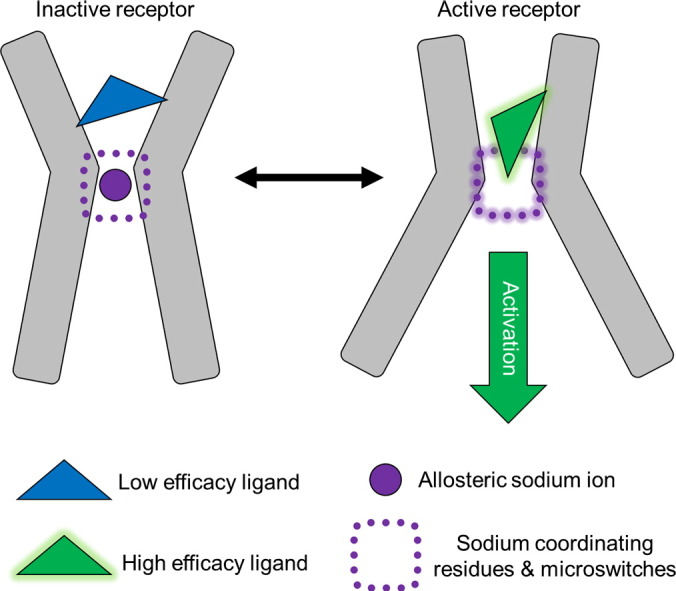

## Introduction

The μ-opioid receptor (MOPr) is a G_i/o_ coupled receptor from the class A G protein-coupled receptor (GPCR) family. It is responsible for both the analgesic and euphoric effects of many opioid drugs [1] and is therefore a protein of very significant clinical and societal importance.

The process of GPCR activation, and particularly the molecular difference between high and low efficacy agonists, is poorly understood. The current consensus is that ligand binding induces changes in residue orientation around the ligand binding pocket, termed micro-switches, that translate to larger rearrangements of the intracellular regions of the receptor, hence promoting engagement with intracellular signalling partners such as G proteins and arrestins [2], [3]. One well-established hallmark of receptor activation is the outward movement of the lower part of transmembrane domain (TM) 6 and the concurrent small inward shifts of TM5 and TM7, thus opening an intracellular cavity in the receptor into which G protein or arrestin can bind [4], [5], [6], [7], [8], [9].

Residues forming a conserved network of polar interactions allosterically connecting the ligand binding site and the intracellular face of MOPr [9], [10], [11] also include those that comprise an allosteric sodium ion binding site [12]. Sodium has been previously described as a negative allosteric modulator of MOPr and other class A GPCRs [13], [14], [15], [16], [17], [18], and a high-resolution X-ray crystal structure of the δ-opioid receptor bound to an antagonist revealed a sodium ion coordinated by conserved residues below the ligand binding pocket [19]. These residues have been proposed to be involved in signal transmission from the ligand binding pocket to the G protein binding site [11], [20], [21], [22], [23]. However, there is limited understanding of the precise nature of this signal transmission through the protein and hence the molecular nature of agonist efficacy.

**Fig. 1:**
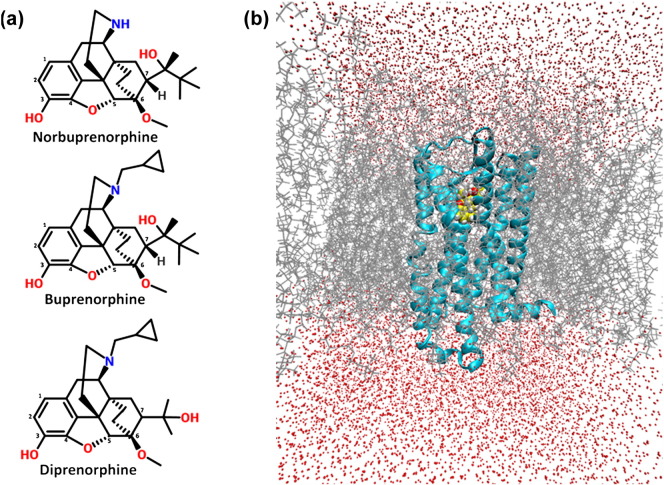
Structurally similar ligands were used in MD simulations bound to MOPr. (a) Structures of the MOPr ligands used in this study, sharing the same morphinan scaffold. Carbons 1–7 are labelled. (b) Model of MOPr (cyan) bound to a ligand, norbuprenorphine (yellow), and embedded in a cholesterol and phospholipid bilayer (grey) solvated in water and NaCl (red), developed from the antagonist-bound crystal structure [31].

The MOPr ligands norbuprenorphine, buprenorphine, and diprenorphine share the same morphinan scaffold (Fig. 1a), and all exhibit affinities for MOPr in the nanomolar range [24], [25] yet display fundamental differences in intrinsic efficacy. Norbuprenorphine, a metabolite of buprenorphine [26], is a full agonist at MOPr, able to activate G proteins and recruit arrestin-3, whilst buprenorphine is a MOPr partial agonist, producing a submaximal activation of G protein, and is unable to induce measurable arrestin-3 recruitment to the receptor [27]. Diprenorphine is a MOPr antagonist [24], that is, it has extremely low or zero efficacy. In this study, we first confirmed the signalling characteristics of these ligands using bioluminescence resonance energy transfer (BRET) assays. Then, these structurally related ligands were used in molecular dynamics (MD) simulations of MOPr to explore ligand binding poses, residue interactions, and MOPr conformations, which may confer the different abilities of these ligands to engage intracellular signalling partners.

## Results

### Agonist-induced G protein activation and arrestin-3 recruitment

**Fig. 2:**
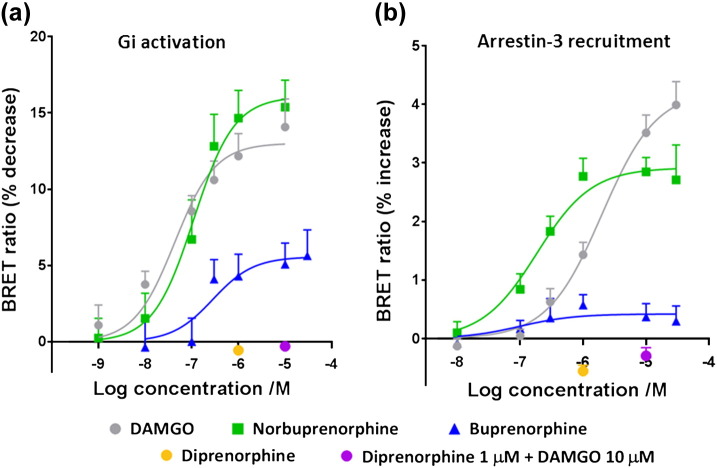
MOPr ligand concentration-response curves from BRET assays. Data obtained by BRET assay in HEK 293 cells (see Materials and Methods) for (a) G_i_ activation and (b) arrestin-3 recruitment to MOPr. Values are mean ± SEM for 3–6 independent experiments.

Human embryonic kidney 293 (HEK 293) cells expressing HA-tagged rat MOPr, Gαi-renilla luciferase (Rluc) II, and Gβγ-green fluorescent protein (GFP) were used to detect dissociation of the Gα and Gβγ subunits upon activation. A decrease in the BRET ratio compared to cells treated with media or 0.01% DMSO alone indicated dissociation, or rearrangement, of these subunits [28]. HEK 293 cells expressing rat MOPr-yellow fluorescent protein (YFP) and arrestin-3-Rluc were used to detect ligand-induced recruitment of arrestin-3 to MOPr. An increase in this BRET ratio indicates arrestin-3 and MOPr coming into closer proximity [29]. Concentration-response curves for G_i_ activation and arrestin-3 recruitment by the MOPr ligands compared to the standard full agonist DAMGO are shown in Fig. 2. EC_50_ and maximum response values for each agonist are listed in Table 1. Compared to DAMGO, norbuprenorphine was a full agonist for G_i_ activation (Fig. 2a) and a potent partial agonist for arrestin-3 recruitment (Fig. 2b), producing approximately 70% of the maximum DAMGO response (Table 1). Norbuprenorphine displayed approximately 10-fold higher potency than DAMGO in the arrestin-3 recruitment and a similar potency as DAMGO for G_i_ activation (Table 1). Buprenorphine was a weak partial agonist for G_i_ activation, producing less than 50% of the maximum response achieved by DAMGO (Fig. 2a). Buprenorphine produced very little response in the arrestin-3 recruitment assay (Fig. 2b). A high concentration (1 μM) of diprenorphine did not produce a response in the G_i_ activation (Fig. 2a) or arrestin-3 recruitment assays (Fig. 2b). Incubation of cells with 1 μM diprenorphine for 10 min prior to the addition of 10 μM DAMGO completely inhibited the DAMGO response for both G_i_ activation and arrestin-3 recruitment (Fig. 2), confirming that diprenorphine is a MOPr antagonist, with extremely low or zero efficacy.

**Table 1:** EC_50_ and maximum response values for MOPr ligands in the BRET assay in HEK 293 cells

Ligand	G_i_ activation		Arrestin-3 recruitment	
	EC_50_ ± SEM (nM)	*E*_max_ (relative to DAMGO)	EC_50_ ± SEM (nM)	*E*_max_ (relative to DAMGO)
DAMGO	42.9 ± 16.9	1.0 ± 0.07	1980 ± 473	1.0 ± 0.06
Norbuprenorphine	110 ± 44.9	1.23 ± 0.12	184 ± 64.5	0.69 ± 0.05
Buprenorphine	283 ± 232	0.43 ± 0.08	– *	0.10 ± 0.03

Data are mean ± SEM for 4–6 independent experiments. Maximum response is expressed relative to DAMGO. * EC_50_ value in the arrestin recruitment assay for buprenorphine was approximately 100 nM.

### Ligand binding poses and residue interactions

**Fig. 3:**
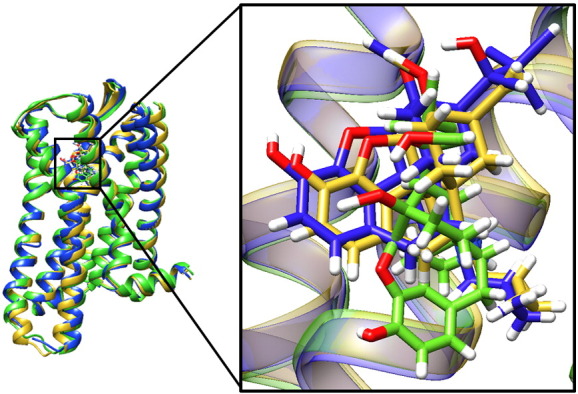
Ligands have different binding poses. Overlay of the average binding poses of MOPr ligands from 1 μs of accelerated MD simulations. Buprenorphine (blue) and diprenorphine (yellow) adopt a position higher in the binding pocket than norbuprenorphine (green).

**Fig. 4:**
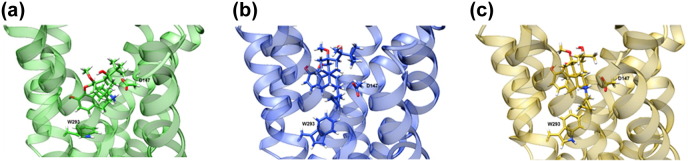
Conformation of the W293^6.48^ toggle switch**.** Representative snapshots of (a) the norbuprenorphine-bound receptor, (b) buprenorphine-bound receptor, and (c) diprenorphine-bound receptor. Showing the ligand, essential residue for opioid binding D147^3.32^, and the different conformations of the conserved W293^6.48^ rotamer toggle switch.

Both conventional MD (cMD) and accelerated MD (aMD) simulations were conducted with murine MOPr (Fig. S6) embedded in a phospholipid and cholesterol bilayer as described in Materials and Methods (Fig. 1b) and bound to norbuprenorphine, buprenorphine, or diprenorphine. aMD is a method of increasing sampling over a short computational time by employing a boost potential to accelerate conformational changes [30]. Moreover, 8 repeats of 125-ns long simulations were performed (with different initial velocities) for each ligand–receptor pair with each method, giving a total of 6 μs of trajectory data. The structure of antagonist β-funaltrexamine (β-FNA) bound to MOPr was used as a template to decide the initial orientation of these ligands [31]. Figures 3 and S1 show the average binding poses after 1 μs of aMD. Despite sharing a common morphinan scaffold and being docked into the same initial binding position, norbuprenorphine adopted a different binding pose to buprenorphine and diprenorphine within the ligand binding pocket. The amine–D147^3.32^ hydrogen bond is essential for opioid binding [32] (superscript numbers follow the Ballesteros–Weinstein numbering system for GPCR residues [33]). Ligands pivoted about this interaction, with norbuprenorphine favouring a position lower (i.e., closer to the intracellular side) in the binding pocket compared to the lower efficacy ligands (Fig. 4). The presence of the cyclopropylmethyl group (Fig. 1a) restricted the ability of buprenorphine and diprenorphine to pivot about the amine–D147^3.32^ interaction; therefore, these ligands favoured a position higher (i.e., nearer to the extracellular side) in the binding pocket. These binding poses were consistent across both 1 μs of cMD and 1 μs of aMD; RMSD plots comparing each ligand to its average binding position show that none of the ligands deviated from the presented average binding pose by more than 1 Å (Fig. S3), and these average binding positions were similar between the aMD and cMD simulations (Fig. S4).

Distances between the heavy atoms of the bound ligand and the residues comprising the binding pocket (Fig. S2) were recorded over the 1-μs simulation time. Residues within 4 Å of each ligand, measured from the average structures, are compared in Table 2, whilst Fig. S5 shows the percentage of simulation time that each residue is within this 4-Å cut-off distance. The MOPr ligands shared some residue interactions; for instance, all three ligands were in contact with D147^3.32^ for at least 80% of the simulation time in both cMD and aMD (Fig. S5) and interacted with H297^6.52^; both residues are known to be essential for opioid ligand binding [32], [34]. In both the aMD and cMD simulations, buprenorphine and diprenorphine were able to interact with K233^5.39^, V236^5.42^, V300^6.55^, and W318^7.35^, due to their location higher in the binding pocket, whilst norbuprenorphine adopted a position further away from these side chains. Similarly, buprenorphine makes contact with Q124^2.60^ and I144^3.29^ due to the carbon-7 side chain (refer to Fig. 1a), whilst diprenorphine lacks this carbon-7 group, and norbuprenorphine sits too low in the receptor binding pocket. Overall, buprenorphine participated in a greater number of ligand–residue interactions in both the aMD and cMD simulations than norbuprenorphine or diprenorphine, which may contribute to the slow dissociation rate of this ligand, compared to norbuprenorphine and diprenorphine [35]. These ligand–residue interactions are similar for both aMD and cMD simulations (Table 2). Differences between the two techniques likely reflect the enhanced sampling by aMD and do not affect the overall interpretation.

**Table 2:**
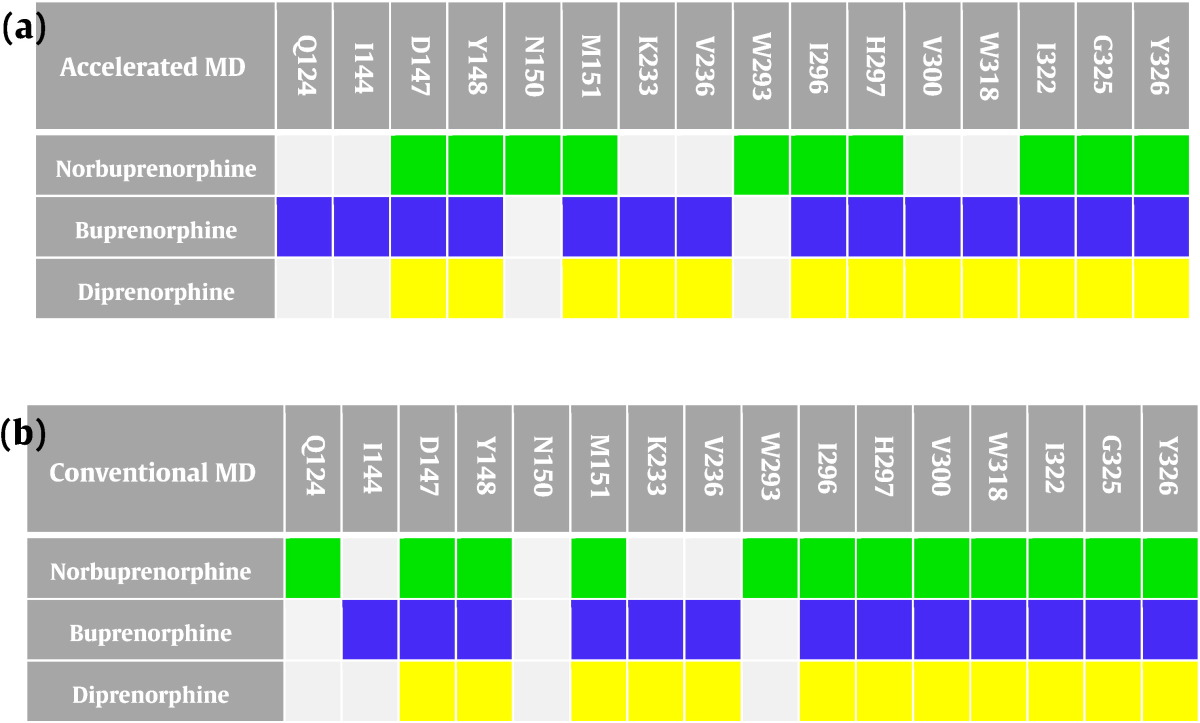
Ligand–residue interactions

Residues within 4 Å of the bound ligand, measured from the average structure of 1-μs simulation data in each case. (a) Accelerated MD; (b) conventional MD.

**Fig. 5:**
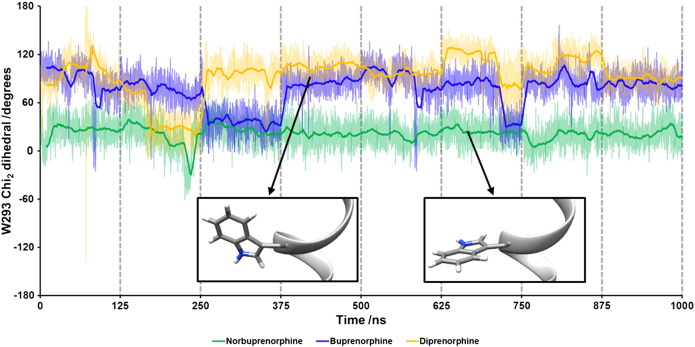
Rotamer angle of W293^6.48^ with each ligand bound, during 1 μs of conventional MD simulations. With norbuprenorphine bound, W293^6.48^ favours a horizontal conformation. Each dataset is plotted as raw data and a running average (solid lines).

It is noteworthy that the high efficacy agonist norbuprenorphine interacted with W293^6.48^ and N150^3.35^, both residues that form part of the allosteric sodium ion pocket below the binding site, whilst the low efficacy agonist and the antagonist did not. Closer inspection of the simulation trajectories revealed that this is due to a small rotation of TM3, causing the N150^3.35^ side chain to fall out of the 4-Å range for buprenorphine and diprenorphine interaction, whilst W293^6.48^ undergoes significant changes in the angle of its side chain (Fig. 4). These rotamer changes can be monitored by plotting the χ2 angle, as shown for the cMD simulation in Fig. 5. With norbuprenorphine bound, the indole ring of W293^6.48^ favours a “horizontal” conformation, parallel to the lipid bilayer, maintaining a χ2 angle of 0–60°, and spanning the base of the ligand binding site. This orientation obstructs the allosteric sodium ion site (Figs. 4a and 5). With buprenorphine (Figs. 4b and 5) or diprenorphine bound (Figs. 4c and 5), the side chain of W293^6.48^ adopts a “vertical” conformation, with the indole ring of the tryptophan perpendicular to the bilayer, favouring a χ2 angle between 80 and 120° and pointing into the ligand binding pocket. Both buprenorphine- and diprenorphine-bound receptors occasionally sample the “horizontal” conformation of W293^6.48^, with buprenorphine more frequently than diprenorphine (Fig. 5).

### Principal component analysis shows distinct helical arrangements

**Fig. 6:**
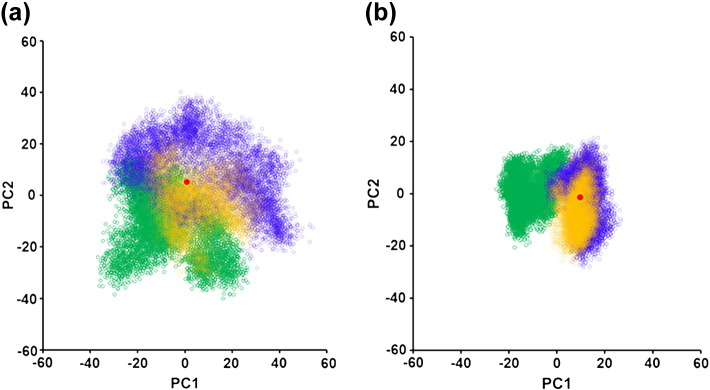
Principal component analysis shows distinct receptor–ligand clusters. Projection of the receptor conformation at each time point onto principal components 1 and 2 (see Materials and Methods). (a) Accelerated MD and (b) conventional MD simulations. The norbuprenorphine-bound receptor is in green, buprenorphine-bound receptor in blue, and diprenorphine-bound receptor in yellow. The conformation of the crystallised β-FNA-bound receptor is projected in red.

Large, high-dimensional datasets, such as that obtained by MD, are difficult to analyse by eye or simple statistics. Principal component (PC) analysis (PCA) is a method of reducing highly dimensional data to the main “principal components”, which account for the most variation [36]. PCA was used in this study to plot subtle changes in receptor conformation and allow the mapping of clusters of conformations explored by the different receptor–ligand complexes. After aligning all trajectories to a set of “core residues” that showed the least movement over the simulation time (Fig. S6a), PCA was performed on the three-dimensional Cartesian coordinates of the alpha carbons of the receptor TMs (Fig. S6b) to avoid variations being masked by the highly dynamic extracellular and intracellular loops and side-chain movements. The receptor conformation at each time point was projected onto PCs 1 and 2, producing clusters of receptor conformations that converge if the helical structures are becoming more similar or diverge if the conformations are different. For the cMD simulations, PCs 1 and 2 account for 28.2% and 11.3% of the variance, respectively. For the aMD, PCs 1 and 2 account for 14.2% and 9.7% of the variance. In both the aMD (Fig. 6a) and cMD (Fig. 6b), the norbuprenorphine-bound MOPr and buprenorphine-bound MOPr form distinct clusters. This indicates different helical arrangements depending on the bound ligand. An overlay of structures extracted from the norbuprenorphine-bound cluster and the buprenorphine-bound cluster is shown in Fig. S7. There are multiple shifts in the positions of several of the transmembrane helices, notably the movement of the extracellular ends of TM1, 2 and 3, and the intracellular end of TM5. The diprenorphine-bound MOPr cluster overlaps somewhat with the buprenorphine-bound cluster, suggesting that the buprenorphine-bound receptor favours a more inactive conformation compared with the norbuprenorphine-bound receptor. The greater spread of the aMD PCA plot (Fig. 6a) shows that the conformational space sampled by aMD is greater than that by the cMD simulations (Fig. 6b), highlighting the value of this technique in increasing sampling over relatively short computational time. Nevertheless, both approaches gave the same overall result.

### Effect of an allosteric sodium ion on ligand binding pose and W293^6.48^ conformation

**Fig. 7:**
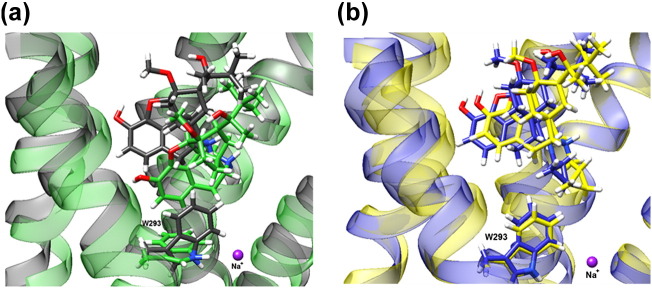
An allosteric sodium ion alters the binding pose of norbuprenorphine. Representative snapshots of the (a) norbuprenorphine-bound receptor and (b) buprenorphine-bound receptor in the presence or absence of an allosteric sodium ion (purple). In the absence of sodium, norbuprenorphine adopts a position deeper in the binding pocket with W293^6.48^ in the horizontal position (green). In the presence of sodium, norbuprenorphine shifts upwards in the binding pocket, and the W293^6.48^ side chain points upwards (dark grey). The binding pose of buprenorphine and the preferred angle of the W293^6.48^ side chain in the absence (blue) and presence (yellow) of sodium do not differ.

During unliganded MOPr simulations, a sodium ion was observed to move from the extracellular space into the receptor pore to occupy the allosteric sodium site (data not shown). The coordinates of this sodium ion were used to set up two repeats of 125-ns cMD and aMD simulations of MOPr bound to norbuprenorphine or buprenorphine in the presence of an allosteric sodium ion, giving a total of 1 μs of trajectory data. Ligand binding poses and the conformation of the W293^6.48^ rotamer in the presence and absence of an allosteric sodium ion are shown in Fig. 7. These conformations were consistent between cMD and aMD simulations. The sodium ion did not leave the allosteric site during the simulation time for either cMD or aMD for either ligand. In the norbuprenorphine-bound simulations (Fig. 7a), the presence of a sodium ion caused the ligand to shift upwards in the binding pocket to occupy a position similar to buprenorphine and diprenorphine. The rotamer of W293^6.48^ switched from a horizontal conformation to the vertical conformation favoured by the buprenorphine- and diprenorphine-bound receptor, pointing into the ligand binding pocket. In the buprenorphine-bound simulations (Fig. 7b), the presence of a sodium ion did not induce significant change in the ligand binding pose or the W293^6.48^ rotamer. The χ2 angle for W293^6.48^ in the presence or absence of sodium for each ligand bound simulation is plotted in Fig. S8. In the presence of sodium, the W293^6.48^ χ2 angle of norbuprenorphine-bound MOPr is maintained at around 80–120°, comparable to the buprenorphine-bound MOPr in both the presence and absence of sodium. RMSD plots indicate that these ligand binding poses in the presence of an allosteric sodium were stable for the entirety of the simulation time (Fig. S9).

**Fig. 8:**
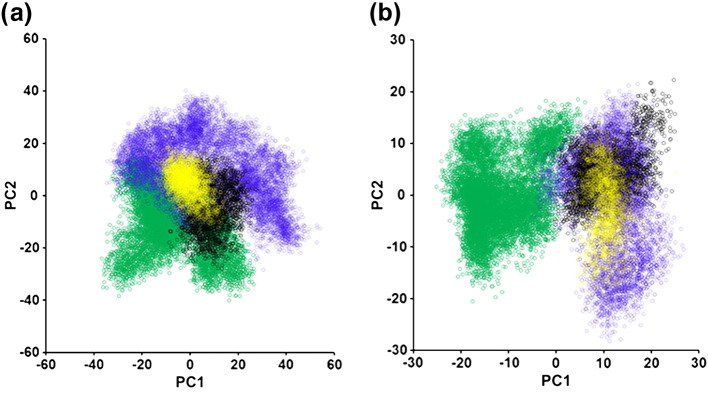
Principal component analysis in the absence and presence of an allosteric sodium ion. Projection of the receptor conformation at each time point onto principal components 1 and 2 (see Materials and Methods). (a) Accelerated MD and (b) conventional MD simulations. The norbuprenorphine-bound receptor in the absence of an allosteric sodium is in green and buprenorphine-bound receptor in the absence of sodium in blue. The norbuprenorphine-bound receptor in the presence of sodium is in black, and the buprenorphine-bound receptor in the presence of sodium is in yellow.

PCA was performed on the receptor TMs as described above. With the sodium ion present, both the buprenorphine and norbuprenorphine clusters overlap with the sodium-free buprenorphine-bound cluster (Fig. 8) and the diprenorphine-bound cluster (Fig. 6). This is consistent for both aMD (Fig. 8a) and cMD simulations (Fig. 8b). Taken together, these PCA plots and the change in ligand binding pose and rotamer of W293^6.48^ suggest that the presence of an allosteric sodium ion causes the norbuprenorphine-bound MOPr to favour an inactive conformation, whilst the buprenorphine-bound MOPr is relatively unchanged.

## Discussion

The results from the BRET assay confirm previous studies characterising norbuprenorphine as a high efficacy agonist capable of recruiting arrestin-3 to MOPr, buprenorphine as a low efficacy agonist with minimal or no arrestin recruitment to the receptor, and diprenorphine as an antagonist for G protein activation and arrestin-3 recruitment, with very low or zero efficacy [24], [27]. Based on some previous studies [27], buprenorphine would be expected to have higher potency in the G protein activation assay; however, higher than expected EC_50_ values for buprenorphine in signalling assays have been reported by others [37]. When compared to the balanced agonist DAMGO, norbuprenorphine appears to display a degree of arrestin bias in these assays. McPherson *et al*. [27] did not report norbuprenorphine exhibiting significant bias between GTPγS binding and arrestin PathHunter assays. This discrepancy may be due to the different assay systems used or that norbuprenorphine could be an arrestin-biased agonist and will be the subject of further investigation.

Both cMD and aMD simulations revealed that these structurally similar ligands adopted distinct binding positions in the ligand binding pocket. The simulated binding poses were maintained over the entire simulation time. Each 125-ns repeat simulation was started from a newly minimised structure and the dynamics initialised with new random velocities and the standard heating protocol. Furthermore, the ligand binding poses for each ligand were similar between repeats of cMD and aMD simulations, giving confidence that the sampling was adequate and the differences in poses seen for the different ligands may be relevant to the physiological situation. Recently reported MD simulations of the β_2_ adrenoceptor revealed ligand–protein interaction fingerprints for a variety of different ligands that correlated with the drug efficacy and the size of the intracellular G protein binding cavity, although the binding positions of the ligands were not greatly different [38].

The presence of a cyclopropylmethyl group on the amine confers a degree of antagonist activity in MOPr ligands [18], [39], [40]. In our simulations, this shared N substituent in buprenorphine and diprenorphine appeared to reduce the ability of the ligand to pivot about the amine–D147^3.32^ interaction so that these low efficacy ligands sat higher in the binding pocket and did not interact with residues in the sodium ion allosteric site and conserved polar network. Using MD simulations of the agonist-bound MOPr, Huang *et al.* [6] have shown that the binding pose of the antagonist BU74 is unstable when the cyclopropylmethyl group was docked into the polar cavity at the base of the binding site and shifts upwards in the binding pocket, suggesting that this ring group cannot stably interact with residues in this polar cavity and in agreement with the data we present here.

The residue W293^6.48^ has previously been identified as a rotamer toggle switch for GPCR activation in the conserved polar network and participating in coordination of a sodium ion [6], [41], [42], [43], [44], [45]**.** The MD simulations described here showed that the full agonist norbuprenorphine came into close contact with this residue, affecting the preferred rotamer of the W293^6.48^ side chain, whereas the low efficacy agonist buprenorphine and antagonist diprenorphine did not. This provides an explanation for the differing efficacies of these ligands. Norbuprenorphine is able to activate this rotamer toggle switch, thus disrupting the allosteric sodium site and allowing MOPr to adopt an active conformation. Buprenorphine and diprenorphine are less able to alter the W293^6.48^ rotamer, so MOPr is less likely to adopt an active conformation. Both the MOPr crystallised with agonist BU72 and the recently described G protein-biased agonist PZM21 also appear to interact with W293^6.48^ [6], [46]. Moreover, MD simulations of MOPr bound to BU72 or another agonist β-fuoxymorphamine show changes in the W293^6.48^ rotamer, suggesting that interaction with this residue is a common mechanism amongst small molecule MOPr agonists [6]. However, these MD simulations with BU72 show the opposite conformation of the W293^6.48^ rotamer to that described here, whilst the χ2 angle of this residue in MD simulations with hydromorphone or morphine bound was different again [47]. This alternative behaviour of W293^6.48^ in the presence of different ligands indicates that receptor activation involves a complex mechanism to give rise to the different helix conformations observed in the present study and cannot be simply explained by a single key residue.

Buprenorphine is a low efficacy agonist [24], [25] but is able to activate MOPr, albeit producing a lower maximum response than higher efficacy agonists. The MD simulations reported here showed buprenorphine adopting an overlapping binding pose, partially overlying PCA clusters, and a similar W293^6.48^ rotamer to the antagonist diprenorphine. This poses the question of how buprenorphine activates MOPr; one possibility is that buprenorphine is sometimes able to engage the W293^6.48^ toggle switch but with lower probability than the high efficacy agonist norbuprenorphine. Indeed, during these MD simulations, buprenorphine did infrequently alter its binding position to overlap with the norbuprenorphine binding pose, with a corresponding change in W293^6 .48^ rotamer (Fig. 5). Another possibility is that buprenorphine is capable of activating MOPr via an alternative mechanism than the W293^6.48^ rotamer. An NMR study of MOPr in the presence and absence of BU72 showed initial agonist-induced conformational changes in helix 8 and intracellular loop 1 preceding the larger movements of TM5 and TM6 [7], although the mechanism of ligand-induced changes in helix 8 and intracellular loop 1 is not yet known. Recently, an MD study comparing MOPr bound to morphine and the G protein-biased ligand TRV-130 revealed alternative allosteric transduction mechanisms between the ligand binding pocket and intracellular G protein binding site for these two drugs [48]. It is possible that buprenorphine can also produce receptor activation via a different mechanism. The PCA plots show the buprenorphine-bound MOPr occupying conformations distinct from both the norbuprenorphine- and diprenorphine-bound receptors (Fig. 6). This may represent a different active conformation stabilised by buprenorphine.

If norbuprenorphine-bound MOPr favours an active conformation, we would predict that the presence of the negative allosteric modulator, sodium, would disrupt this conformation. Indeed, we found that whilst an allosteric sodium ion did not significantly alter the binding pose, W293^6.48^ rotamer, and PCA clustering of the buprenorphine-bound MOPr, the presence of a sodium ion did disrupt the norbuprenorphine-bound MOPr, causing it to occupy a more inactive conformation. Other MD studies that started with a sodium ion occupying the allosteric site of the active-like adenosine A_2A_ receptor have observed either the sodium leaving the binding pocket or the sodium ion remaining bound and the GPCR adopting an inactive conformation whilst agonist binding is destabilised [49]. Here, the sodium ion did not leave the receptor pore, but the bound ligand and MOPr adopted inactive conformations. This perhaps reflects the relatively short simulation time and that the simulations were started from an already inactive receptor structure, therefore favouring retention of the sodium ion in the allosteric pocket and destabilisation of the agonist binding pocket.

We propose that the distinct receptor conformations captured by the PCA reflect the differing ligand efficacies at the MOPr. Although the BRET experiments suggested that norbuprenorphine may display some bias towards arrestin recruitment, this is yet to be validated in further experiments, and so, the ability to discriminate between an active MOPr conformation and an arrestin-biased conformation by MD simulations is yet to be achieved. In order to characterise receptor conformations that favour G protein or arrestin signalling, a larger set of structurally diverse test compounds, some displaying a strong bias for G protein or arrestin signalling, would be required.

In conclusion, this study has identified molecular differences between structurally related MOPr ligands that correlate with their different intrinsic agonist efficacies. MD simulations showed the high efficacy agonist norbuprenorphine favouring an alternative position in the binding pocket to the low efficacy agonist buprenorphine and the antagonist diprenorphine, without the overlap of the morphinan scaffold. This different pose allowed norbuprenorphine to interact with the W293^6.48^ toggle switch, important for GPCR activation and part of the allosteric sodium site. Importantly, the MOPr TMs occupy distinct sets of conformations with a different ligand bound. Together, these results suggest that small changes in the ligand binding pose and ligand–residue interactions lead to global conformational changes in the MOPr helices and induce different receptor conformations. These alternative helical conformations identified in this MD study may confer different abilities of MOPr to activate intracellular signalling partners.

## Materials and Methods

### Cell culture and transfection

HEK 293 cells were cultured at 37 °C in Dulbecco's modified Eagle's medium (DMEM) supplemented with 10% foetal bovine serum and penicillin/streptomycin. Cells were seeded onto 10-cm dishes and grown to 80% confluence before transfection. Cells were transfected with a 1:1 ratio of rat MOPr conjugated to YFP and arrestin tagged with Rluc or with a 1:1:1 ratio of HA-tagged rat MOPr, Gαi-RlucII, and Gβγ-GFP, 24 h before assay, using jetPEI DNA transfection reagent (Polyplus).

### BRET assay

Immediately prior to assay, cells were resuspended in clear DMEM and then transferred to a 96-well plate at 90 μl per well. DAMGO, buprenorphine, and norbuprenorphine dissolved in water; diprenorphine dissolved in DMSO; and coelenterazine h and coelenterazine 400a dissolved in methanol were diluted in DMEM media to the required concentration. The final DMSO concentration for diprenorphine and controls was 0.01%. The BRET assay was performed on the FLUOstar Omega microplate reader. Drugs and coelenterazine were added at time 0, and the luminescence was measured over 2 min (G protein activation) or 10 min (arrestin recruitment). The ratio of the light emitted by YFP or GFP to the light emitted by Rluc was used to calculate the BRET ratio. To determine whether diprenorphine can antagonise the DAMGO response, we incubated the cells with 1 μM diprenorphine for 10 min, prior to the addition of 10 μM DAMGO, and the assay was performed as described above. All treatments were performed in duplicate, and the average response was taken. The assay was repeated in 3–6 independent experiments. Data were expressed as the percentage increase (arrestin recruitment) or decrease (G protein activation) in the BRET ratio from control cells treated with media or media + 0.01% DMSO, and analyses and curve fitting were performed in GraphPad Prism version 7. All ligands were compared to DAMGO as the standard high efficacy agonist at MOPr.

### System preparation for MD

The X-ray crystal structure of antagonist-bound MOPr (PDB: 4DKL) [31] was obtained from the Protein Data Bank and prepared in Insight II. Ligands and the T4 lysozyme were removed, and a loop search was performed to find a homologous loop to model in the missing intracellular loop 3. A loop was selected by visual inspection and the residues changed to the correct mouse MOPr sequence (Fig. S6). The side chains were inspected for clashes and bonds rotated where necessary. The receptor was embedded in a POPC:POPE:cholesterol lipid bilayer at a 5:5:1 ratio using the replacement method, and the simulation box (initial dimensions: 90, 110, 90 Å) solvated with TIP3P water and 0.15 M NaCl using the CHARMM-GUI software [50]. The antagonist β-FNA shares a morphinan scaffold with buprenorphine, norbuprenorphine, and diprenorphine. The binding pose of β-FNA in the crystal structure was used as a template to determine the initial orientation of the ligands for MD simulation. Ligands were parameterised using Antechamber and the general Amber force field [51]. All ligands were protonated at the amine to allow interaction with D147^3.32^. Amber parameter topology and coordinate files were prepared in LEaP.

### MD simulations and analysis

Structures were minimised over 10,000 steps; then, the system was heated under constant volume and pressure with lipids restrained, from 0 K to 100 K over 5 ps, and then from 100 K to 310 K over 100 ps. We performed 10 rounds of 500-ps equilibration under constant pressure to equilibrate the periodic box dimensions. Each simulation was run for 125 ns under the Amber ff14SB and Lipid14 force fields [52], [53]. Temperature and pressure were controlled using the Langevin thermostat and the anisotropic Berendsen barostat, with a 2-fs time step and trajectories written every 100 ps. Simulations were run for a total of 1 μs for each receptor–ligand complex in a series of 8 × 125 ns parallel steps, with newly minimised structures and new random velocities for each simulation, under both cMD and aMD [30]. aMD uses an external boost potential to accelerate conformational changes, allowing for increased sampling over the same amount of computational time as cMD. This technique allows protein conformational changes to be investigated, which would otherwise not be accessible over the short computing time available. Parameters for the aMD were calculated as described in Kappel *et al.* [54]. Trajectories were visualised in VMD, and the analysis was performed using cpptraj [55]. Trajectories were aligned to a set of “core” residues that showed the least amount of movement across the simulations (Fig. S6a) to avoid including general translation and rotation of the protein in the analysis, before RMSD and PCA calculations were performed. The covariance matrix was calculated and diagonalised using cpptraj, and the PCs were obtained by mapping the three-dimensional Cartesian coordinates of the alpha carbons of the TMs of all trajectories (Fig. S6b). Each frame of the simulated trajectories was mapped onto PC1 and PC2 to produce a plot visualising the conformational space occupied by the receptor at each time point. Images of ligand binding poses and side-chain orientations were prepared in Chimera [56].

### Simulations with an allosteric sodium ion

Simulations were also performed with MOPr in the absence of any ligand. During these unliganded simulations, a sodium ion was observed to enter the receptor pore from the extracellular space and occupy the allosteric sodium site (data not shown). The position of this sodium ion was in agreement with the published high-resolution structure of the antagonist-bound δ-opioid receptor [19]. The coordinates of this allosteric sodium ion in the unliganded simulation were used to place a sodium ion into the receptor pore of MOPr bound to buprenorphine or norbuprenorphine. Two independent 125ns simulations were run, as described above, for each ligand, under both aMD and cMD simulations, giving a total of 500 ns of trajectory data for each ligand–sodium complex.

## Appendix A Supplementary Data

Supplementary figures Image 2
