# Supplementary material for: Drug Binding Poses Relate Structure with Efficacy in the μ Opioid Receptor
Source: J Mol Biol. 2017 Jun 16;429(12):1840–51. doi: 10.1016/j.jmb.2017.05.009 (PMC5472181; doi:10.1016/j.jmb.2017.05.009)

**Supplementary figures**


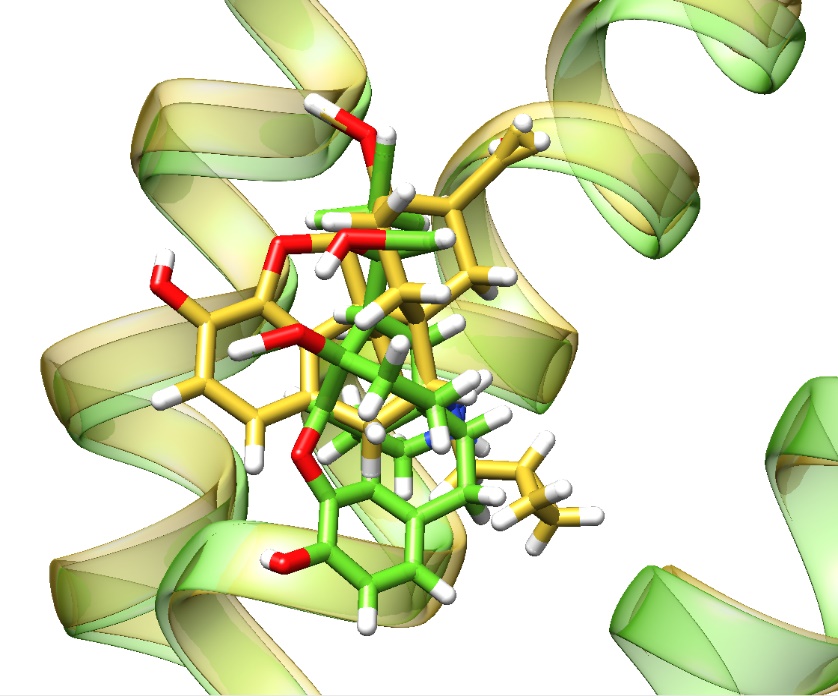

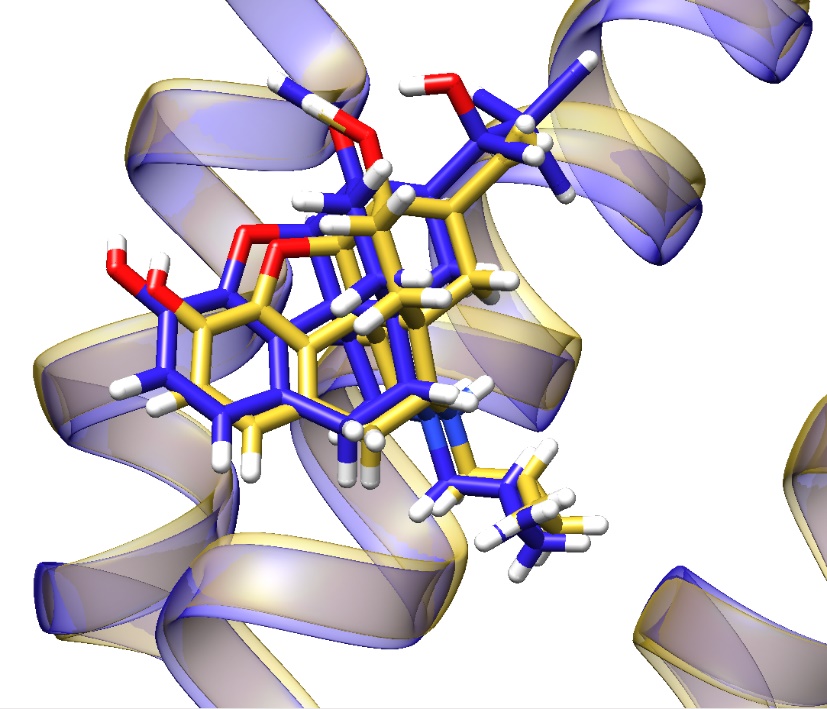

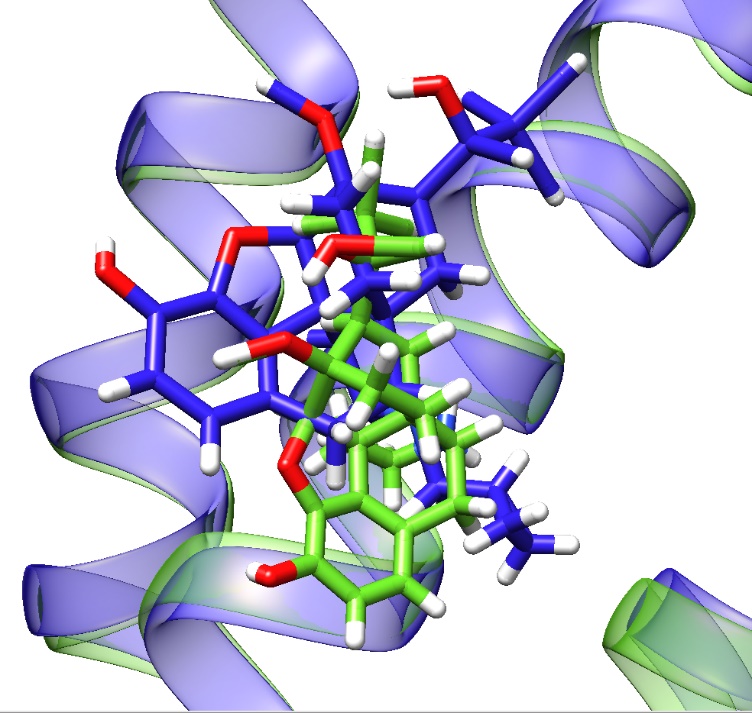


**Supplementary Fig. 1. Ligand binding poses.** Overlay of the average binding poses of MOPr ligands from 1 µs of accelerated MD simulations. Buprenorphine (blue) and diprenorphine (yellow) adopt a position higher in the binding pocket than norbuprenorphine (green).

**(a)**

**(b)**

**(c)**


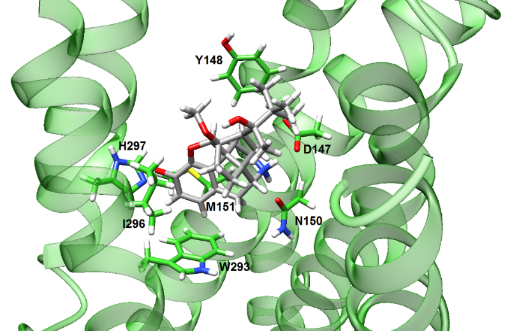

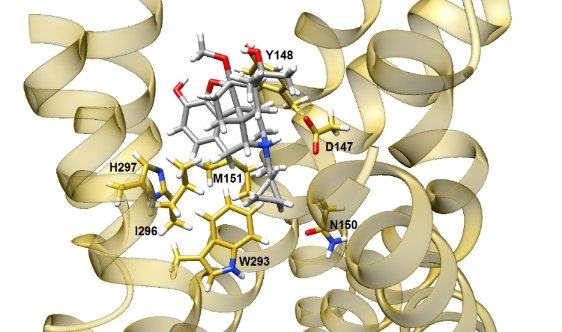

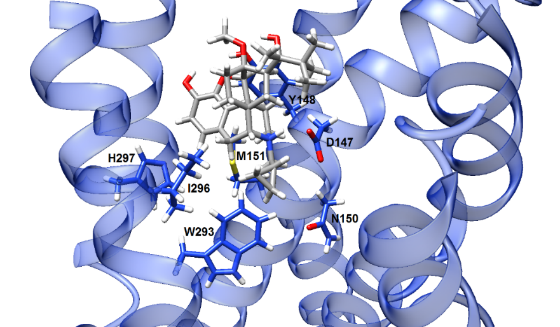


**(a)**

**(b)**

**(c)**

**Supplementary Fig. 2. Ligand binding poses and key residues.** Representative snapshots of the binding poses of: (a) norbuprenorphine; (b) buprenorphine; (c) diprenorphine. Structures are viewed looking through the lipid bilayer at the side of the receptor. Key interacting residues forming the ligand binding pocket are depicted.

**Supplementary Fig. 3.** **RMSD plots show ligand binding poses are stable.**

RMSD plots of the heavy atoms of each ligand compared to each ligand’s average binding pose. The binding positions are stable over 1 µs of cMD and aMD.

**Conventional MD**

**Accelerated MD**

**Norbuprenorphine**

**Buprenorphine**

**Diprenorphine**


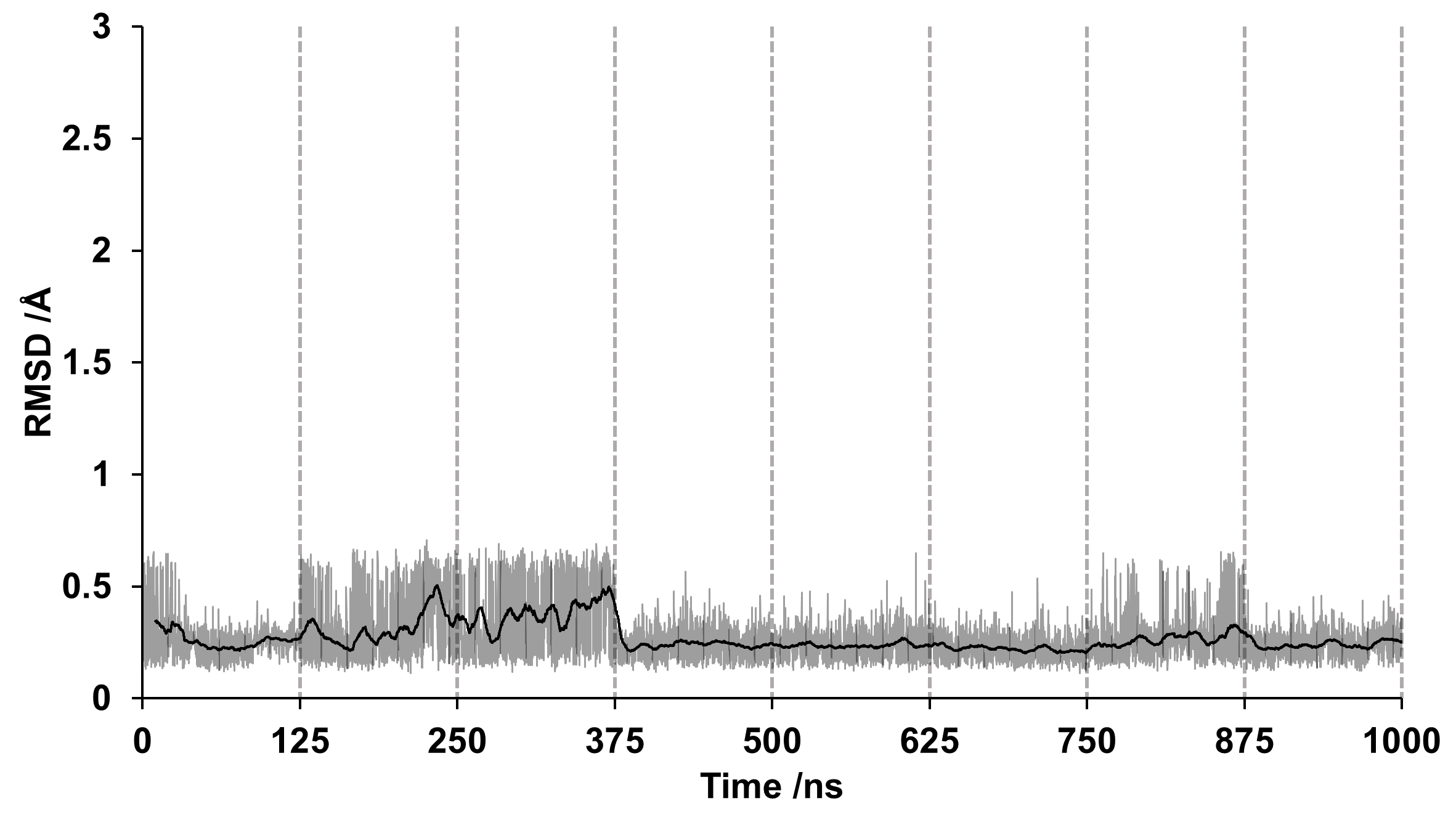

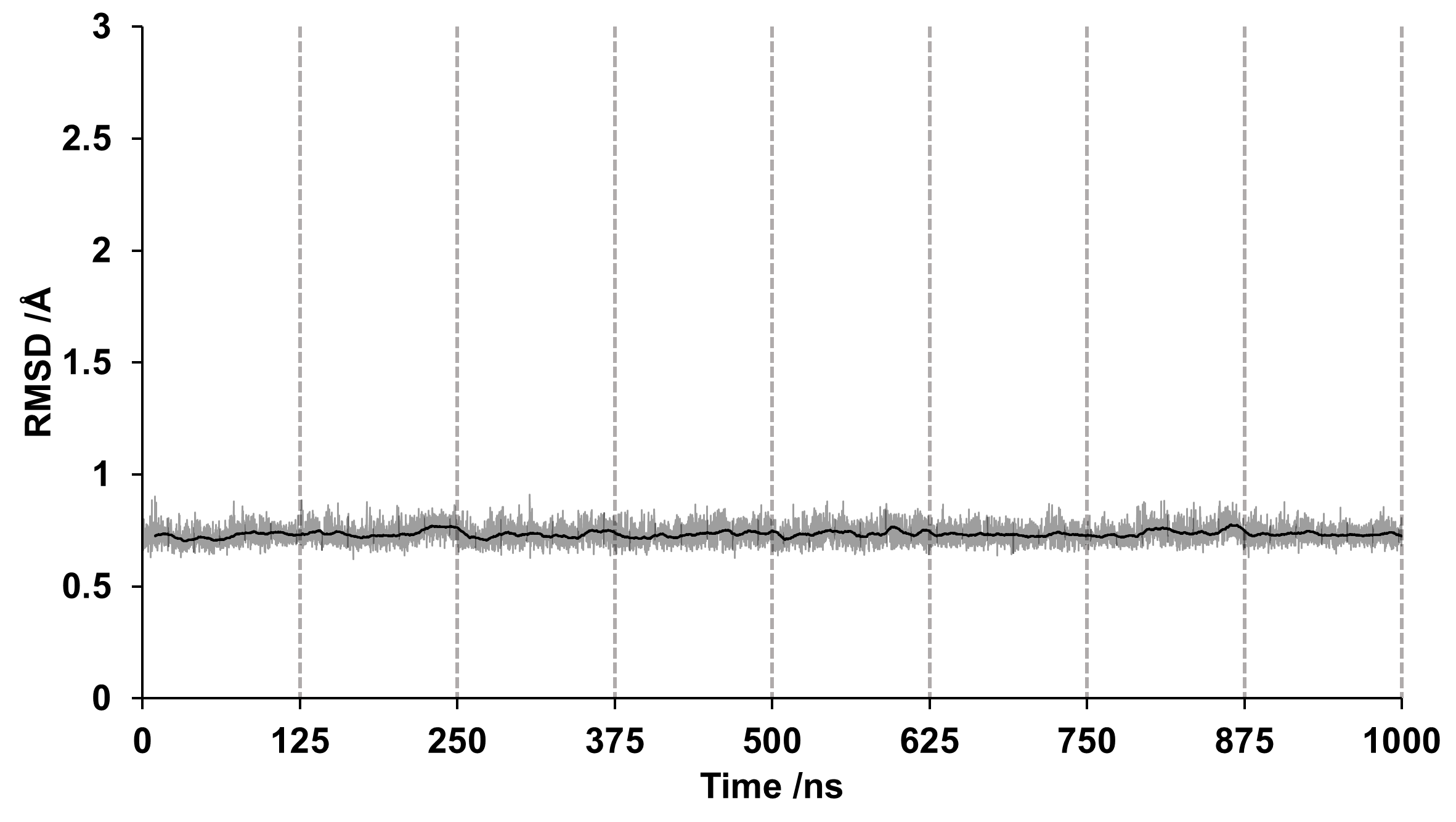

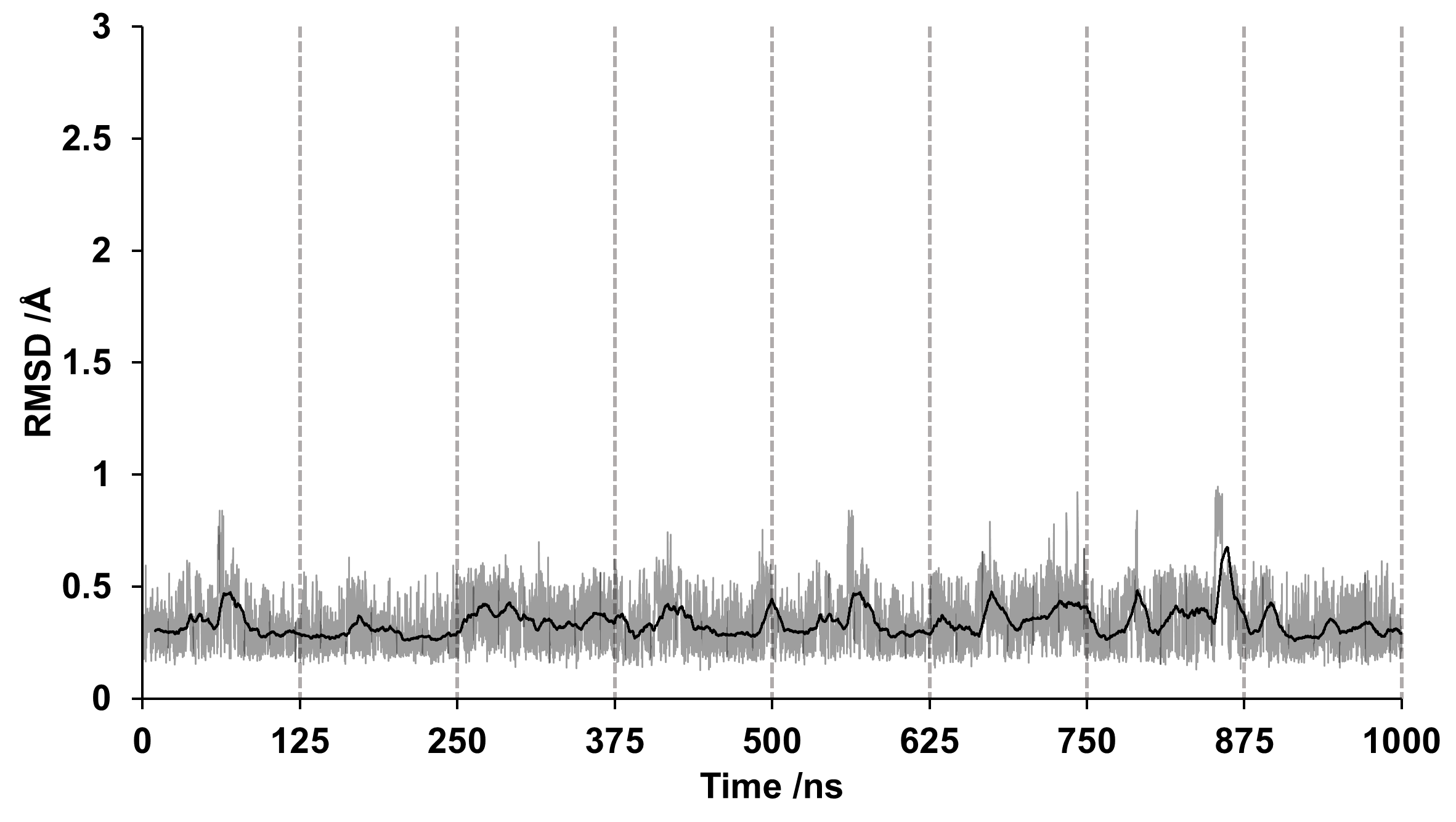

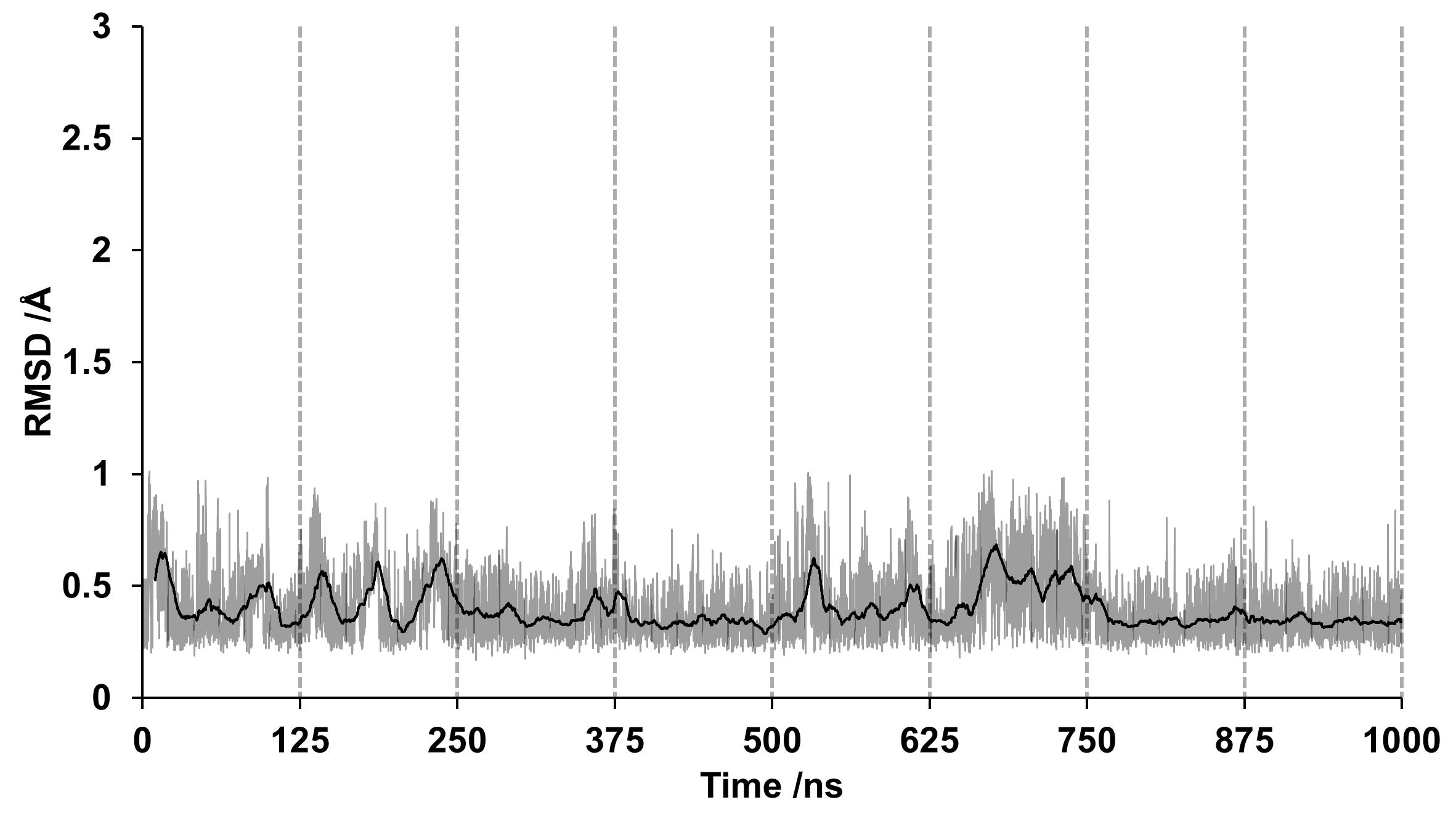

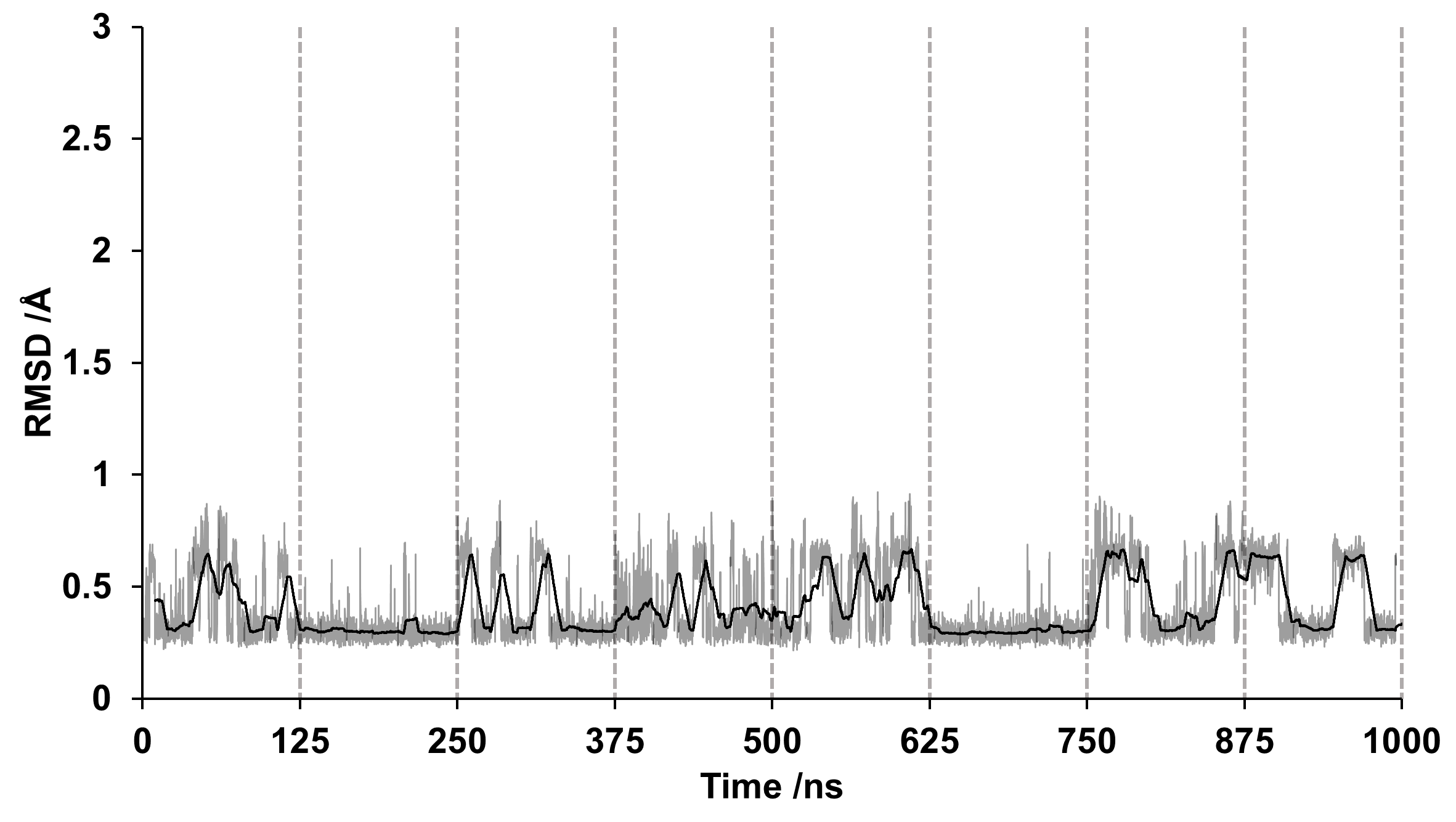

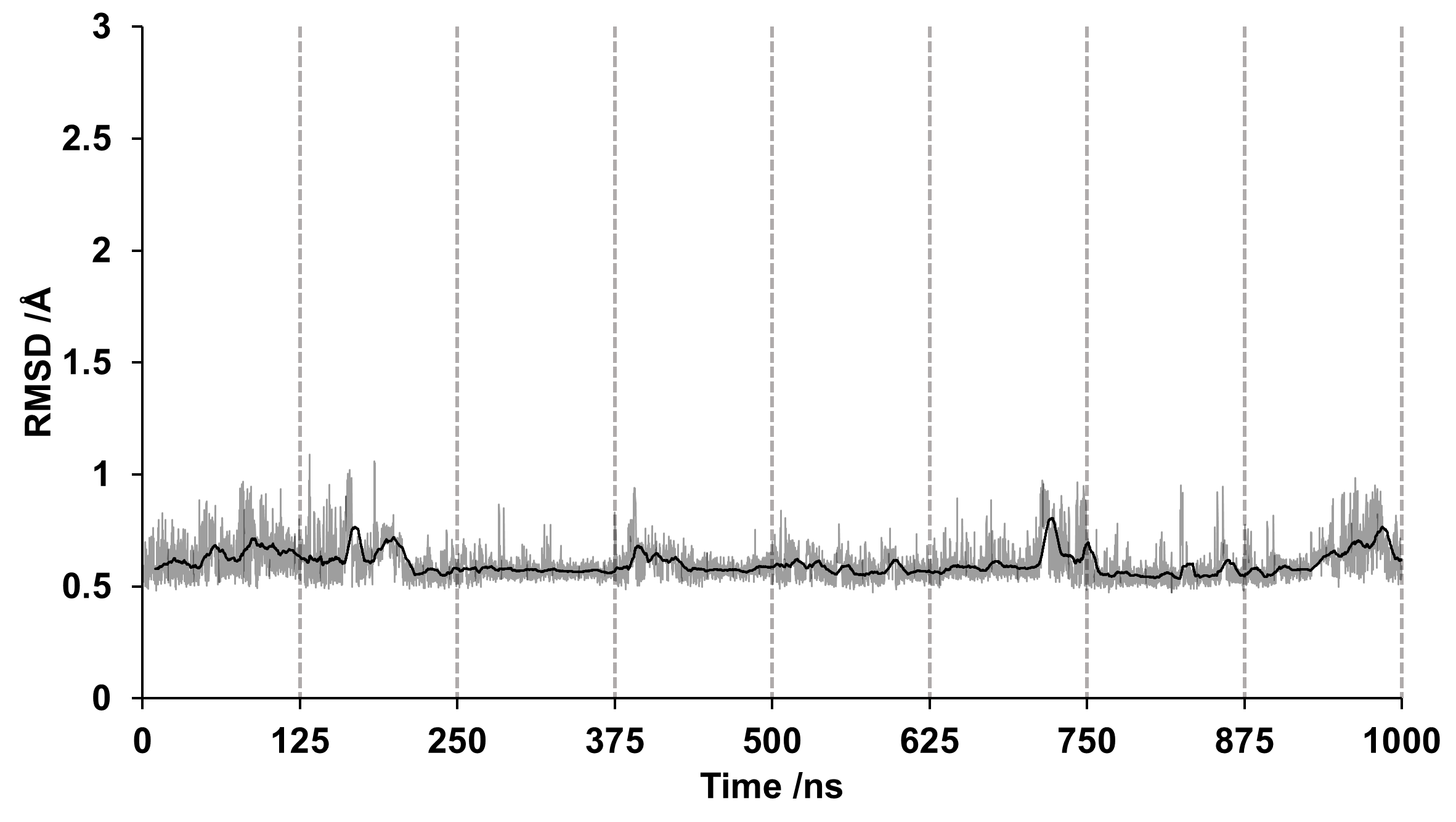

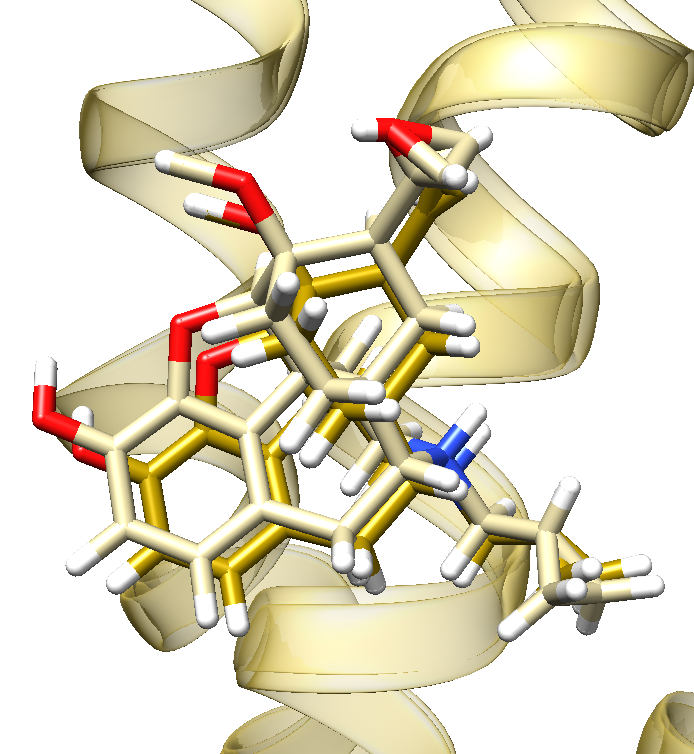

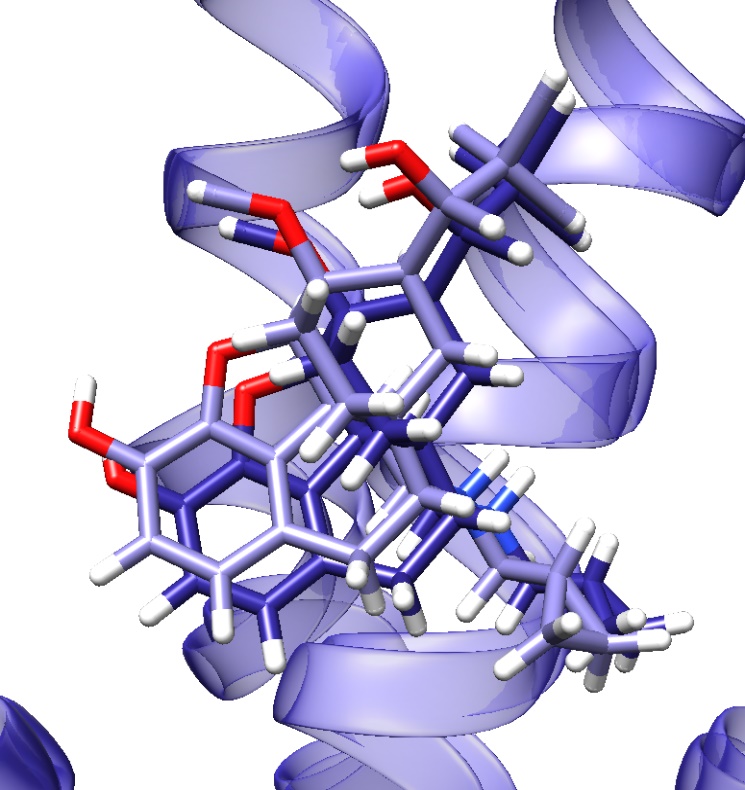

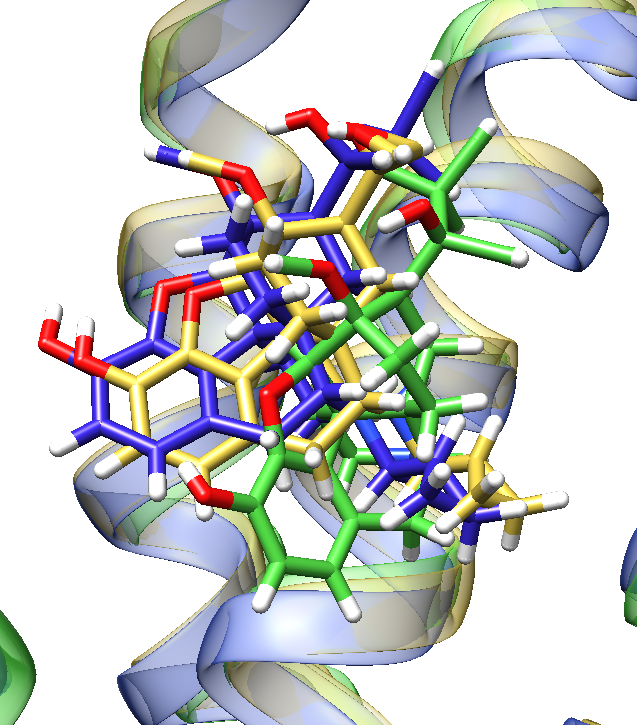

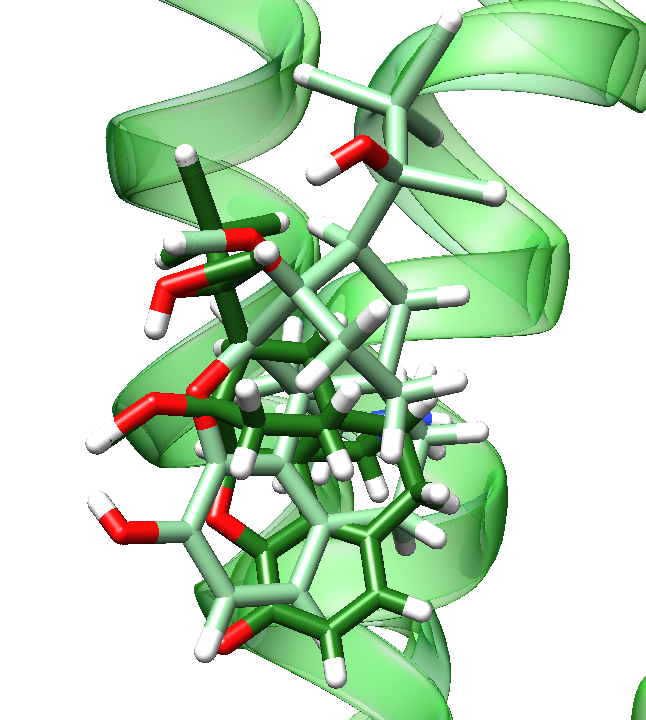


**(a)**

**(b)**

**(c)**

**(d)**

**Supplementary Fig. 4. The average binding poses for aMD and cMD are very similar.**

(a) Overlay of the average binding poses for each ligand from 1 μs cMD simulations. (b) Average poses for norbuprenorphine from the cMD (light green) and aMD (dark green) simulations. (c) Average binding poses for buprenorphine from cMD (light blue) and aMD (dark blue) simulations. (d) Average poses for diprenorphine from the cMD (light yellow) and aMD (dark yellow) simulations.


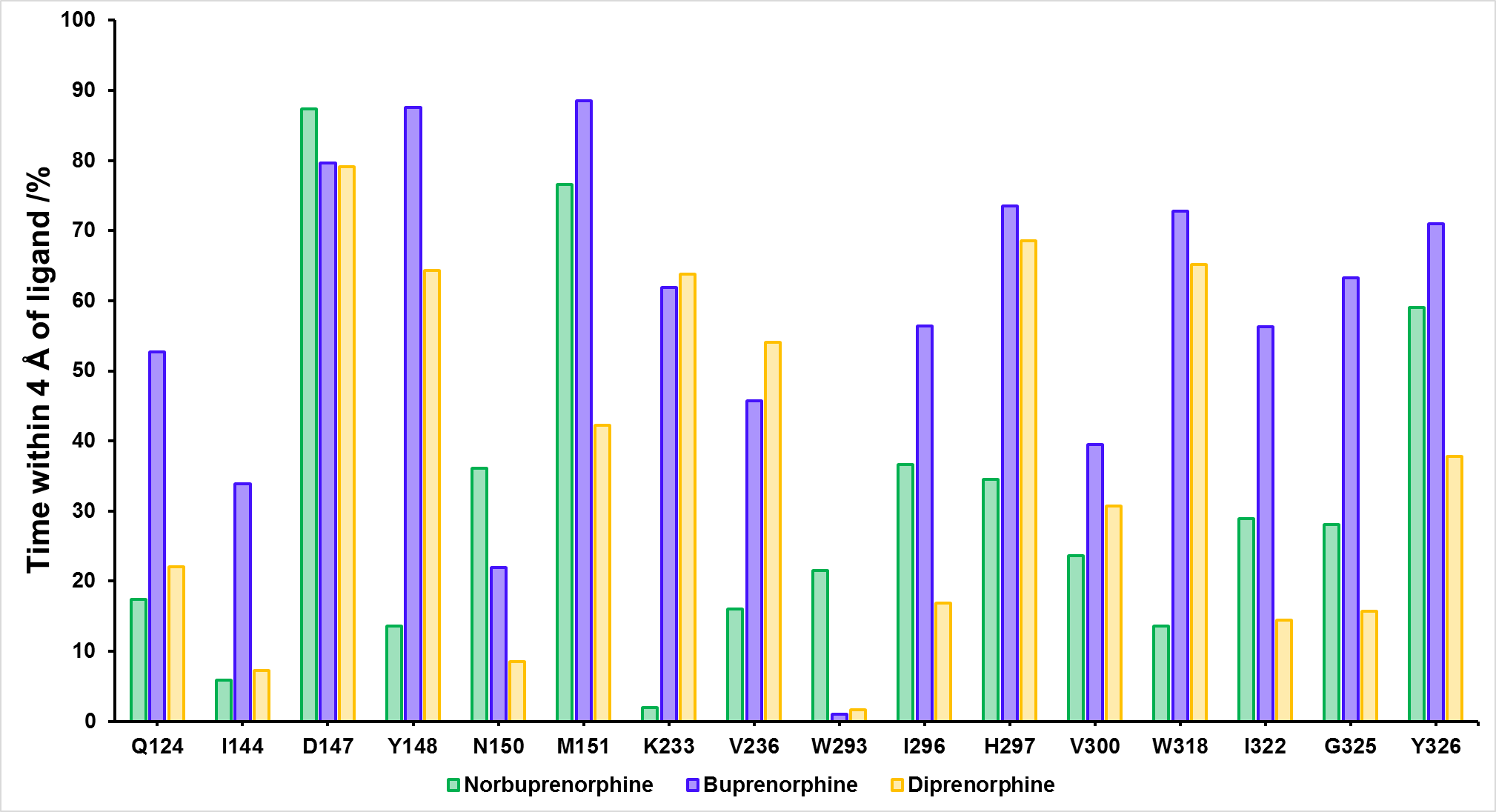

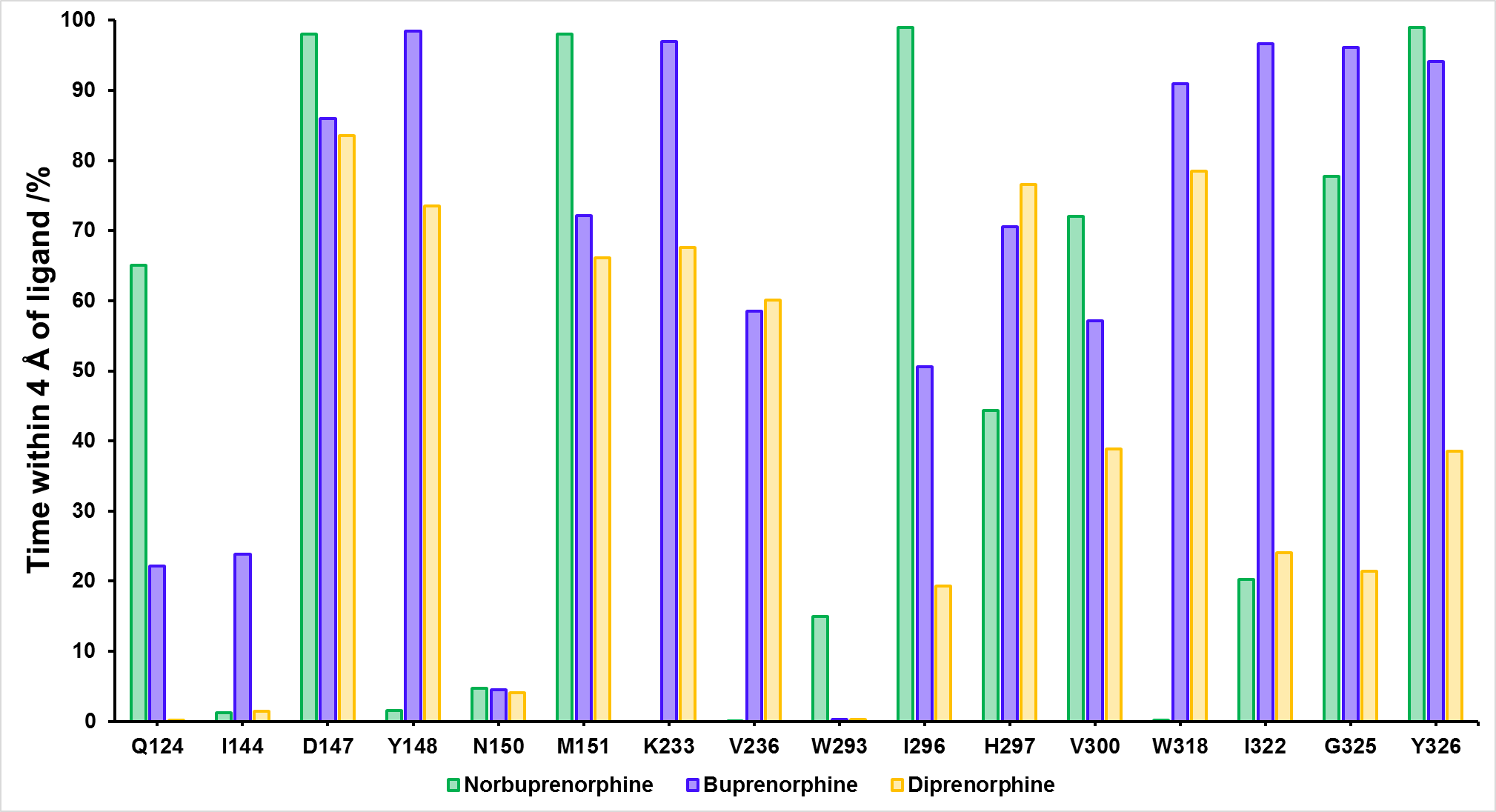


**(a)**

**(b)**

**Supplementary Fig. 5. Ligand-residue interactions as a percentage of simulation time.** Percentage of the simulation time that residues are found within 4 Angstroms of the bound ligand for (a) 1 µs of accelerated MD and (b) 1 µs of conventional MD.


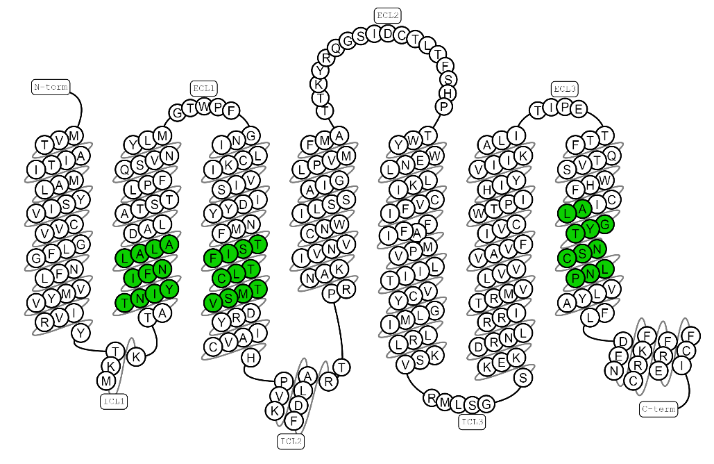

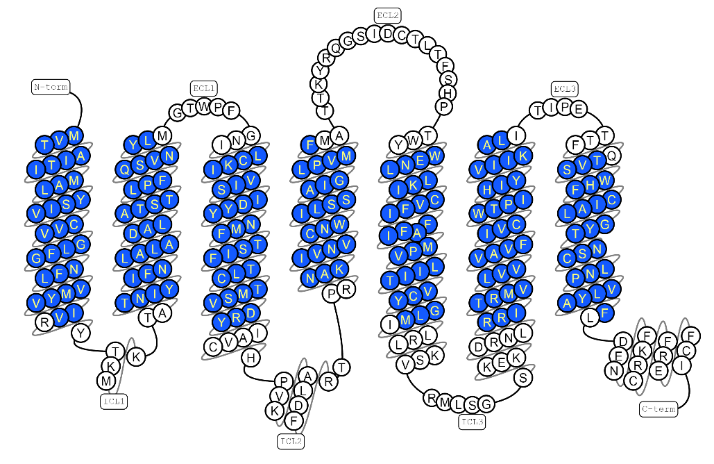


**Supplementary Fig. 6. Snake plots of the murine MOPr sequence used in the MD simulations.**

(a) The core residues to which the trajectories are aligned prior to PCA are shown in green. (b) The transmembrane domain regions for the PCA are shown in blue.

**(a)**

**(b)**

**Extracellular side**

**Intracellular side**

**Supplementary Fig. 7. Structures extracted from the PCA plot for 1 μs cMD simulations.**

Red arrows indicate shifts of the helices between the structure extracted from the buprenorphine-bound cluster (blue) and the structure extracted from the norbuprenorphine-bound cluster (green).


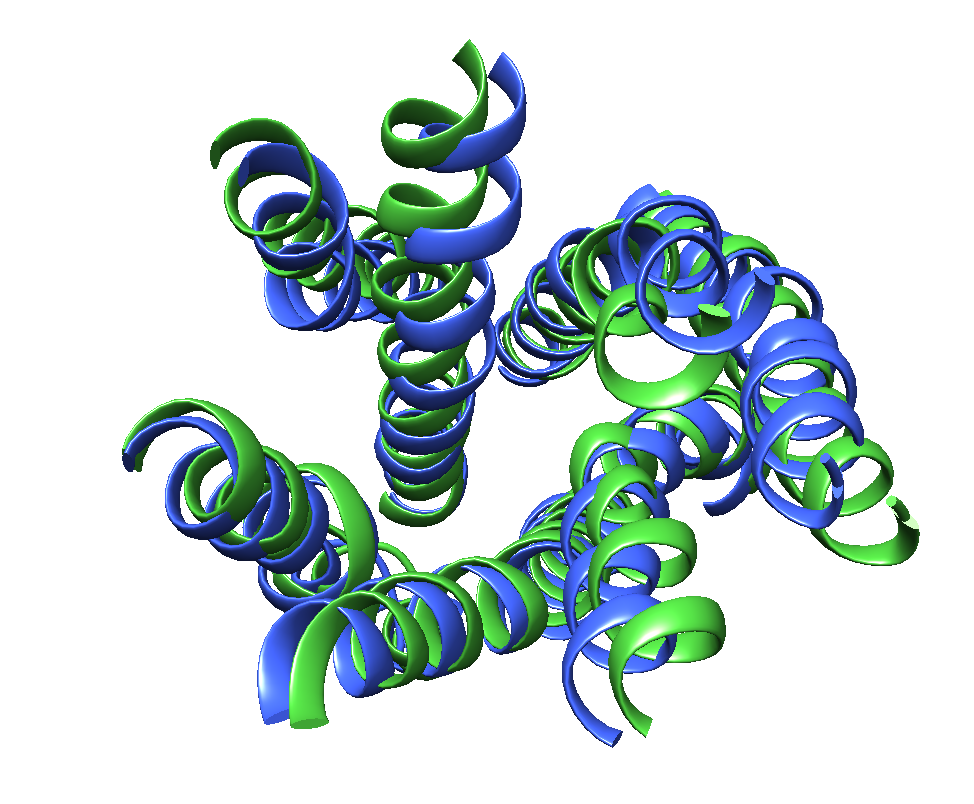


**TM3**

**TM4**

**TM5**

**TM6**

**TM7**

**TM1**

**TM2**


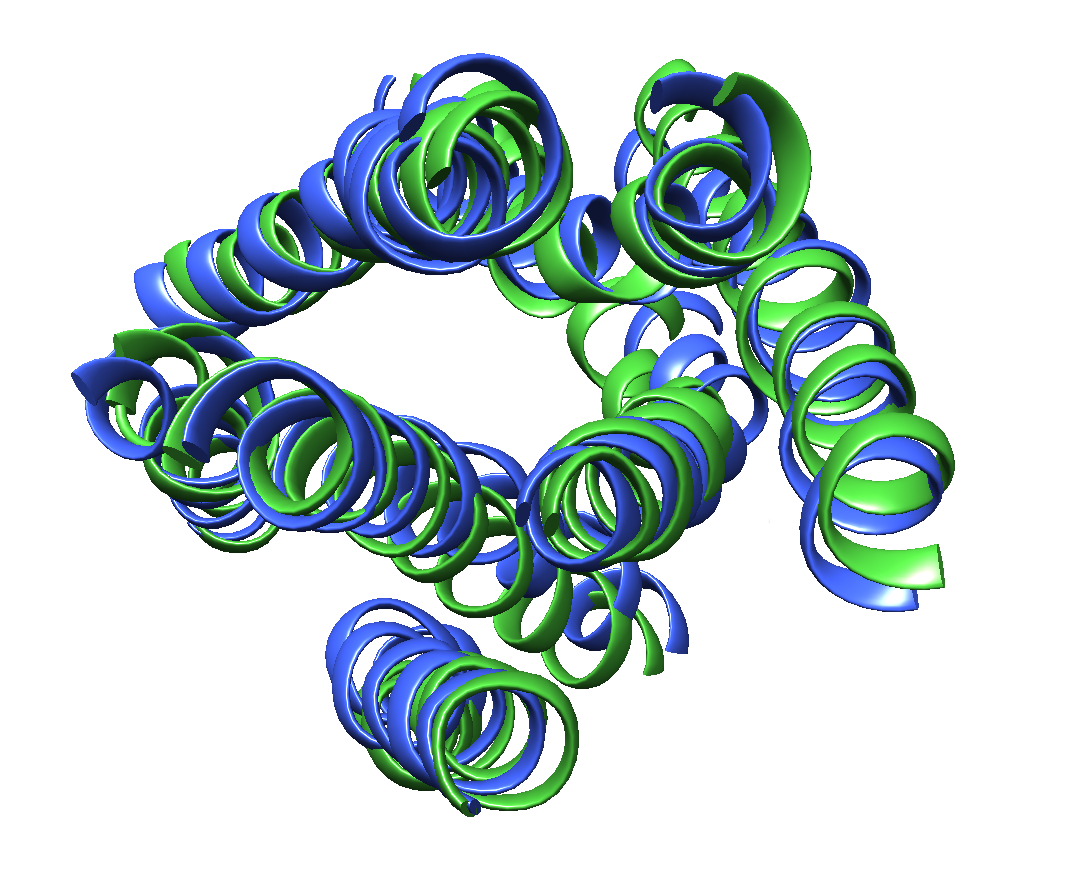


**TM5**

**TM6**

**TM7**

**TM1**

**TM2**

**TM3**

**TM4**

**Supplementary Fig. 8. W293^6.48^ conformation in the presence and absence of an allosteric sodium.** Rotamer angle of W293^6.48^ over 250 ns conventional MD with norbuprenorphine or buprenorphine bound, in the presence or absence of an allosteric sodium ion. Presence of an allosteric sodium causes W293^6.48^ to adopt a similar rotamer angle to the buprenorphine- and diprenorphine-bound simulations. Each dataset is plotted as raw data and a running average (solid lines).


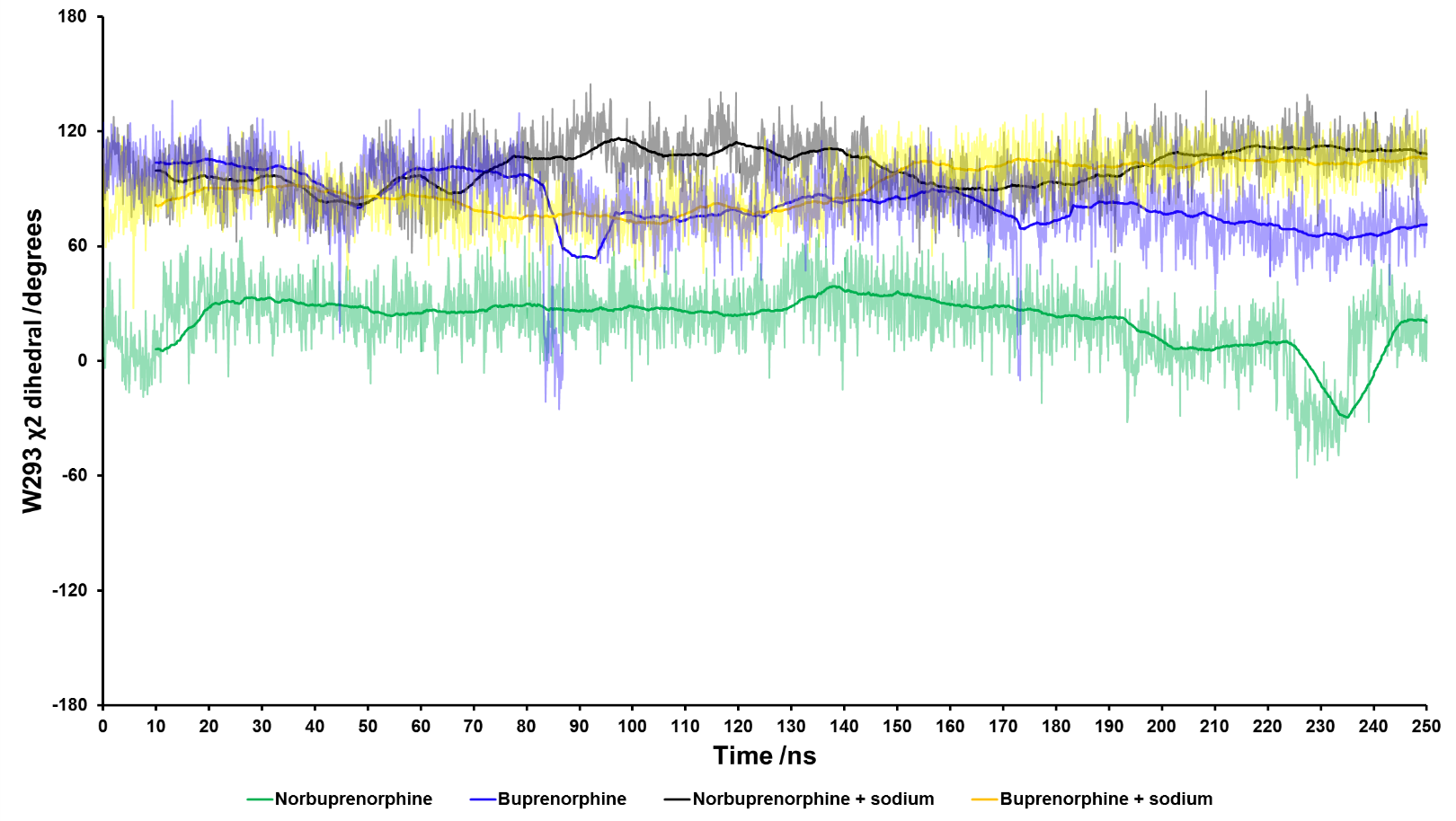

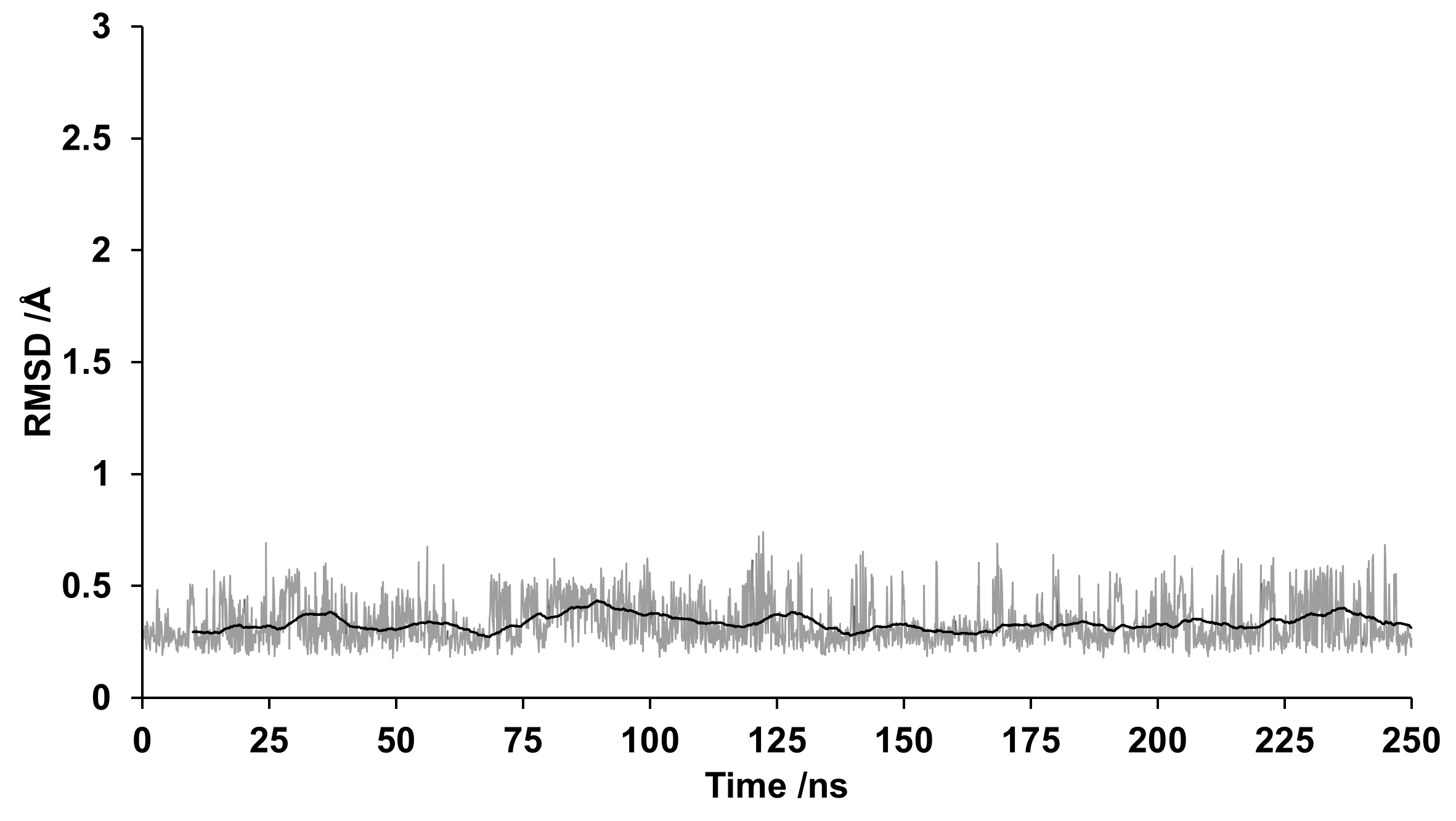

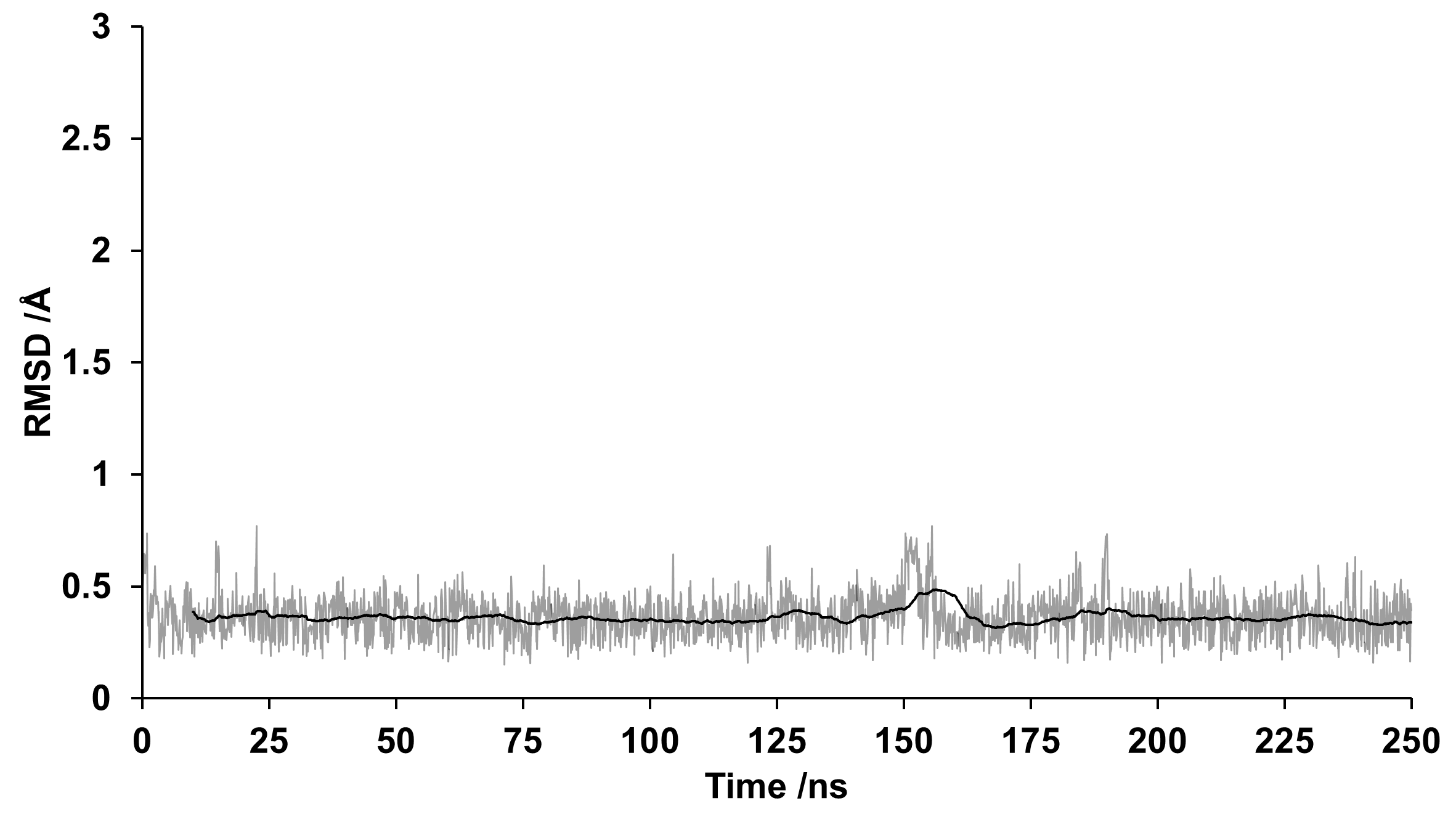


**Norbuprenorphine**

**Buprenorphine**

**Conventional MD**

**Accelerated MD**

**Supplementary Fig. 9.** **RMSD plots show ligand binding poses in the presence of an allosteric sodium ion are stable.**

RMSD plots of the heavy atoms of each ligand compared to each ligand’s average binding pose. The binding positions are stable over 250 ns of cMD and aMD.


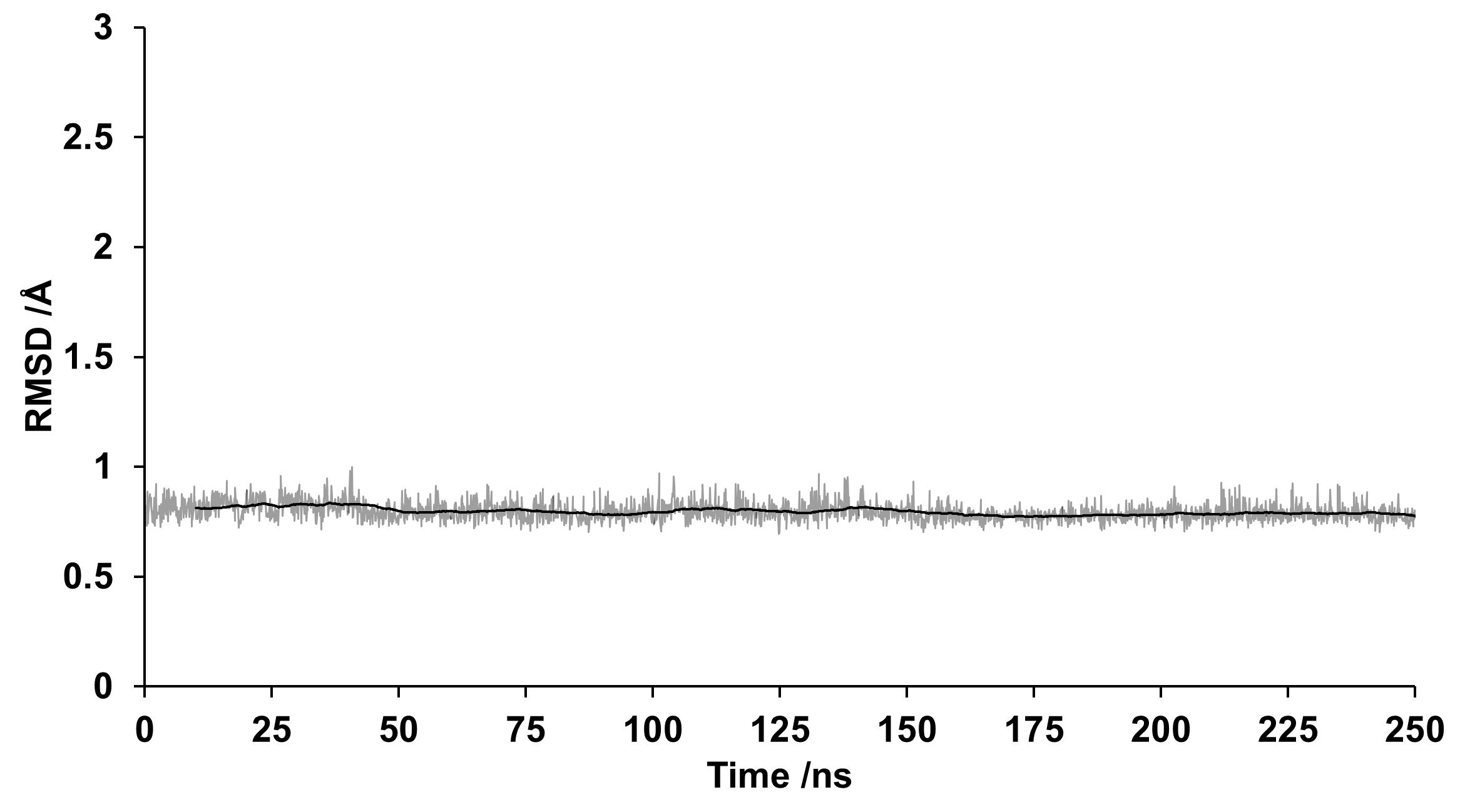

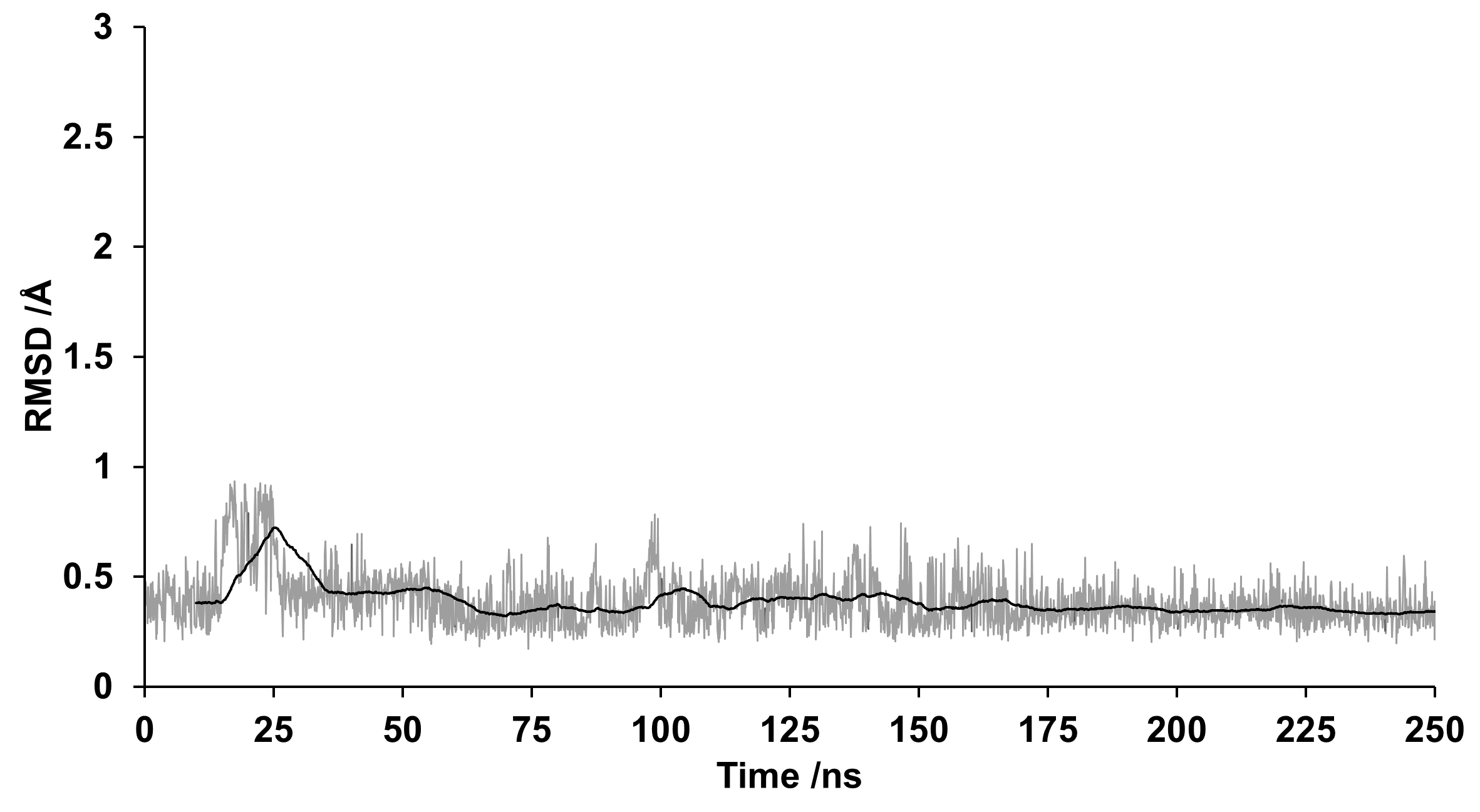

Supplement: Supplementary file 1 — Supplementary figures [file mmc1.docx]
